# circZNF148 Drives Glucose Metabolism Reprogramming to Enhance Metastasis and Immune Evasion via HK1 Stabilization in Triple‐Negative Breast Cancer

**DOI:** 10.1002/advs.77030

**Published:** 2026-08-07

**Authors:** Yuhan Jin, Wenjing Zhao, Lei Wang, Jianing Wang, Keying Zhao, Dan Luo, Yifei Wang, Yuxia Yang, Jingze Yang, Yaming Li, Tong Chen, Dianwen Han, Zekun Wang, Xiaoyan Li, Bing Chen, Lijuan Wang, Yiran Liang, Qifeng Yang

**Affiliations:** ^1^ Department of Breast Surgery General Surgery Qilu Hospital of Shandong University Jinan Shandong People's Republic of China; ^2^ Biological Resource Center Qilu Hospital of Shandong University Jinan Shandong People's Republic of China; ^3^ Department of Breast Surgery General Surgery The Affiliated Taian City Central Hospital of Qingdao University Taian Shandong People's Republic of China; ^4^ Research Institute of Breast Cancer Shandong University Jinan Shandong People's Republic of China

**Keywords:** circZNF148, glycolysis, immune evasion, lactylation, triple‐negative breast cancer

## Abstract

Breast cancer is the leading malignancy among women worldwide, with triple‐negative breast cancer (TNBC) representing the most aggressive subtype. Accumulating evidence highlights glucose metabolism reprogramming as a critical driver of TNBC progression and immune evasion, yet the underlying molecular mechanism remains poorly defined. Using circRNA sequencing in both glucose metabolism‐stressed TNBC cells and lung metastasis models, we identified circZNF148 as a consistently upregulated circular RNA that promotes tumor progression and immune evasion by enhancing glycolysis. Mechanistically, circZNF148 scaffolds the interaction between hexokinase 1 (HK1) and the deubiquitinase adaptor ADRM1, suppressing K11‐linked ubiquitination and proteasomal degradation of HK1, thereby stabilizing the enzyme and sustaining glycolytic flux. Elevated lactate production subsequently impairs CD8^+^ T cell function, reducing IFN‐γ and TNF‐α secretion, diminishing cytotoxicity, and promoting exhaustion. Concurrently, increased intracellular lactate drives lysine lactylation of PD‐L1, which enhances its protein stability and membrane localization, thus linking metabolic state to immune checkpoint regulation. Clinically, circZNF148 is significantly upregulated in TNBC tissues, correlating with advanced stage and poor prognosis. Collectively, our findings establish the circZNF148‐HK1‐lactate axis as a critical mediator of metabolic adaptation and immune evasion in TNBC. Targeting this pathway offers a promising strategy to overcome therapeutic resistance and improve outcomes in TNBC.

## Graphical abstract

CircZNF148 stabilizes HK1 through deubiquitinase recruitment, thereby enhancing glycolysis and lactate production. Elevated lactate promotes PD‑L1 lactylation and membrane accumulation while suppressing CD8^+^ T‐cell cytotoxicity, collectively facilitating immune evasion and malignant progression in TNBC. This metabolic‐immune checkpoint axis may represent a promising therapeutic target in aggressive breast cancer.

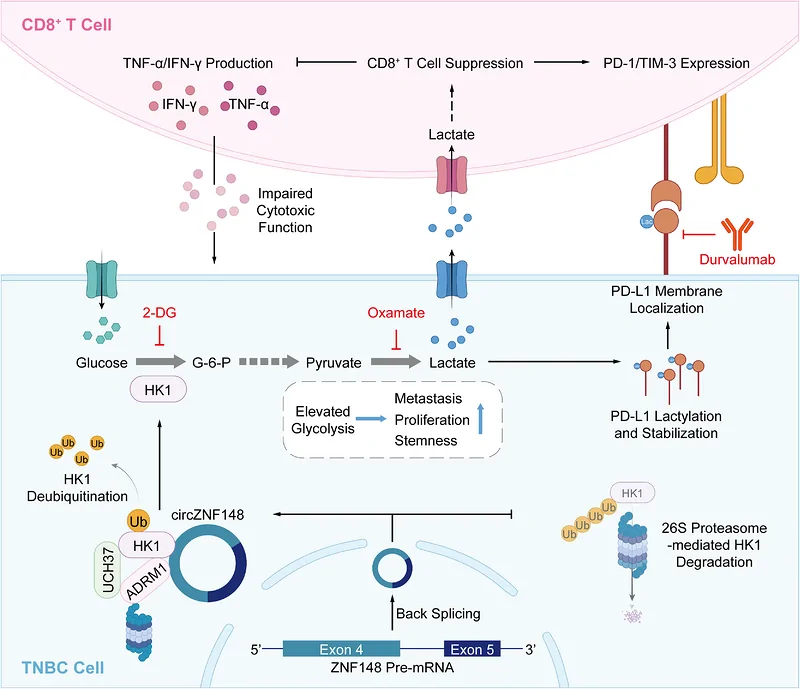

## 1 Introduction

Breast cancer remains the most frequently diagnosed malignancy among women worldwide, accounting for approximately 32% of all female cancers and ranking second in cancer‐related mortality [1]. Among its molecular subtypes, triple‐negative breast cancer (TNBC), defined by the absence of estrogen receptor (ER), progesterone receptor (PR), and human epidermal growth factor receptor 2 (HER2) expression, represents the most aggressive and therapeutically challenging subtype. Characterized by high heterogeneity, early relapse, chemoresistance, and a strong propensity for visceral metastases, TNBC is associated with limited treatment options and poor prognosis despite advances in systemic therapy [2, 3, 4]. Notably, metastatic TNBC is associated with markedly reduced survival, underscoring the urgent need to elucidate its regulatory mechanisms and identify novel therapeutic targets.

A hallmark of TNBC is metabolic reprogramming, particularly the upregulation of glycolytic enzymes such as hexokinase (HK), which drives enhanced glycolytic flux [5]. This glycolytic dependency is further amplified in metastatic TNBC cells [6], suggesting a functional link between metabolic adaptation and metastatic fitness [7, 8]. Beyond supporting bioenergetic demands for tumor proliferation and metastasis, this metabolic shift actively shapes an immunosuppressive tumor microenvironment (TME). Enhanced glycolysis in tumor cells leads to local glucose depletion and lactate accumulation, which impair CD8^+^ T cell function by reducing glycolytic capacity, suppressing cytotoxicity, and limiting effector lymphocyte infiltration [9, 10]. Additionally, the programmed cell death protein 1 (PD‐1)/programmed death‐ligand 1 (PD‐L1) signaling axis plays a predominant role in mediating immune evasion in TNBC. PD‐L1, regulated by multiple oncogenic and inflammatory signals, is more highly expressed in TNBC than in other breast cancer subtypes [11], and blockade of this pathway has become a cornerstone of first‐line therapy for PD‐L1‐positive metastatic disease [12]. Nevertheless, patients with metastatic TNBC exhibit limited or transient responses to immune checkpoint inhibitors (ICIs) [13, 14], highlighting the critical need to uncover the underlying mechanisms. Intriguingly, accumulating evidence reveals that metabolic intermediates can directly modulate immune checkpoints, further coupling tumor metabolism to immune evasion. Lactate, a major byproduct of glycolysis, not only acidifies the TME, but also serves as a substrate for lysine lactylation (Kla), a recently discovered post‐translational modification that stabilizes PD‐L1 and enhances its immune‐inhibitory activity [15]. This finding suggests that glycolytic metabolism may amplify PD‐L1‐mediated immunosuppression, providing a potential explanation for the limited efficacy of ICIs in metabolically active tumors.

Circular RNAs (circRNAs), a class of covalently closed, stable non‐coding RNAs, have emerged as critical regulators of cancer biology [16]. Recent studies indicate that certain circRNAs are differentially expressed under metabolic stress and contribute to tumor progression and therapy resistance [17, 18, 19]. However, whether circRNAs participate in the crosstalk between metabolic reprogramming and immune evasion in TNBC remains largely unexplored.

In this study, we identify circZNF148 as a stress‐responsive and metastasis‐associated circRNA that correlates with poor prognosis in breast cancer. We demonstrate that circZNF148 functions as a molecular scaffold to stabilize HK1 through interaction with the deubiquitinase adaptor ADRM1, thereby enhancing glycolytic flux and lactate production. Elevated lactate levels impair CD8^+^ T cell effector function and drive lysine lactylation of PD‐L1, increasing its stability and membrane expression. Collectively, our findings reveal a previously unrecognized circZNF148‐HK1‐lactate axis that couples metabolic adaptation to immune evasion in TNBC, offering a promising dual‐targeting strategy to suppress metastasis and restore anti‐tumor immunity.

## 2 Materials and Methods

### 2.1 Ethics Statement and Human Tissue Samples

Approval for all experimental protocols was secured through the Institutional Review Board of Qilu Hospital, Shandong University, with written informed consent prospectively obtained from all participants. Resected specimens underwent independent histopathological validation through triple‐blinded consensus review by board‐certified surgical pathologists, followed by immediate cryopreservation at −80 °C.

### 2.2 Cell Culture and Transfection

Human breast cancer cell lines (MDA‐MB‐231 and MDA‐MB‐468) and HEK293T cells were originally obtained from the American Type Culture Collection (ATCC). All cell lines were routinely authenticated by short tandem repeat (STR) profiling and confirmed to be free of mycoplasma contamination. Cells were maintained in Dulbecco's Modified Eagle Medium (DMEM, MAC GENE TECHNOLOGY LTD, Beijing, China) supplemented with 10% fetal bovine serum (FBS) and 1% penicillin–streptomycin at 37°C in a 5% CO_2_ humidified incubator.

For routine cell culture, breast cancer cells were maintained in high‐glucose DMEM (Cat #CM15019, containing 4.5 g/L glucose). For low‑glucose experiments, cells were washed with PBS and subsequently cultured in low‐glucose DMEM (Cat# CM10014, containing 1 g/L D‐glucose) for 24 h. Both media contain 3.7 g/L NaHCO_3_ as the primary pH buffer. The pH of both media was measured after 24 h of incubation under 5% CO_2_ at 37°C. To ensure that the observed metabolic and molecular changes under low‐glucose conditions were not secondary to stress‐induced cell death, cell viability was monitored using the MTT assay. These assays confirmed that low‐glucose exposure did not induce significant cytotoxicity, with cells maintaining a high relative viability (>90% compared to high‐glucose controls) after 24 h of treatment.

Small interfering RNAs (siRNAs) were purchased from GenePharma (Shanghai, China). The sequences of siRNAs were listed in Table S1. Transfections were performed using JetPRIME reagent (Polyplus, France) according to the manufacturer's protocol.

### 2.3 Plasmid Construction

To overexpress circZNF148, the genomic sequence spanning exons 4 and 5 of ZNF148 was amplified and cloned into the pLCDH‐ciR vector. For HK1 and ADRM1 overexpression, the full‐length coding sequences were cloned into pCMV and pcDNA3.1 vectors, respectively. Additionally, various fragments of HK1 and ADRM1 were cloned into the pCMV vector with a 3× Flag tag and the pcDNA3.1 vector with a Myc tag, respectively.

### 2.4 RNA Extraction and Sequencing Analysis

Total RNA was extracted using TRIzol reagent (Invitrogen, USA) according to the manufacturer's instructions. RNA sequencing and bioinformatic analysis were performed by Cloud‐Seq Biotech (Shanghai, China). Differential gene expression analysis was conducted using the Bioconductor package edgeR.

### 2.5 RNase R and Actinomycin D Treatment

To assess RNA circularity and stability, 2 µg of total RNA was incubated with or without 3 U RNase R at 37°C for 20 min, followed by enzyme inactivation at 70°C for 10 min. For RNA stability assays, cells were treated with 5 µg/mL actinomycin D (ActD, Sigma, USA) and harvested at specified time points prior to RNA extraction. The levels of circRNAs were detected by qRT‐PCR.

### 2.6 RNA Isolation and Quantitative Real‐Time PCR

For circRNA and mRNA detection, cDNA was synthesized using the PrimeScript RT Reagent Kit (Takara, Shiga, Japan). qRT‐PCR was carried out using Hieff qPCR SYBR Green Master Mix (Yeasen Biotechnology, China). The relative expression levels were calculated using the 2^−^^ΔΔCt^ method, with β‐actin serving as the endogenous control for circRNA/mRNA and U6 for miRNA. The primer sequences are listed in Table S2.

### 2.7 Western Blot Analysis

Cells were lysed in RIPA buffer (Beyotime, China) supplemented with PMSF and NaF, and protein concentrations were determined using the BCA Protein Assay Kit (Millipore, USA). Equal amounts of protein were separated by 10% SDS–PAGE, transferred to 0.22 µm PVDF membranes (Millipore), and blocked in 5% nonfat milk for 1 h at room temperature. Membranes were incubated with primary antibodies overnight at 4°C, followed by HRP‐conjugated secondary antibodies for 1 h at room temperature. Signals were visualized using enhanced chemiluminescence (ECL). The antibodies were listed in Table S3.

### 2.8 Subcellular Fractionation

Cytoplasmic and nuclear RNA were isolated using the PARIS Kit (Invitrogen, USA) following the manufacturer's protocol.

### 2.9 Immunofluorescence

Cells were fixed with 4% paraformaldehyde (PFA), permeabilized with 0.5% Triton X‐100, and blocked with 10% goat serum for 1 h at room temperature. After blocking, the cells were incubated with specific primary antibodies overnight at 4°C. The following day, the cells were incubated with appropriate secondary antibodies for 2 h in the dark, followed by nuclear counterstaining with DAPI for 15 min at room temperature. Finally, the stained cells were visualized and imaged using a fluorescence microscope. The antibodies were listed in Table S3.

### 2.10 Fluorescence In Situ Hybridization (FISH)

A Cy3‐labeled probe targeting circZNF148 was synthesized by GenePharma (Shanghai, China). FISH assays were performed using a fluorescence in situ hybridization kit following standard procedures. Briefly, breast cancer cells were fixed with 4% paraformaldehyde, permeabilized with 0.5% Triton X‐100, and hybridized with the circRNA probe overnight at 37°C in the dark, followed by nuclear counterstaining with DAPI for 15 min at room temperature. Finally, the stained cells were visualized and imaged using a fluorescence microscope.

### 2.11 In Situ Hybridization (ISH)

ISH was performed using the Enhanced Sensitive ISH Detection Kit I (BOSTER, China) according to the manufacturer's protocol. A digoxigenin‐labeled probe targeting circZNF148 (sequence: AAGTATAACTGCTTTTGCGGGAGAACGTT) was synthesized by GenePharma (Shanghai, China). Tissue sections were fixed with 4% paraformaldehyde for 15 min, incubated with 30% H_2_O_2_/methanol solution, pre‐hybridized at 37°C for 4 h, and hybridized with the probe overnight at 37°C. On the following day, sections were sequentially incubated with blocking reagent, biotinylated anti‐digoxigenin antibody, SABC, and biotinylated HRP. DAB was used for chromogenic detection, and hematoxylin staining was performed for nuclear counterstaining. Images were captured under a bright‐field microscope.

### 2.12 Flow Cytometry (FCM) Analysis

Apoptosis was assessed using the Annexin V Apoptosis Detection Kit I (BD Pharmingen) following the manufacturer's protocol. Transfected cells were washed twice with PBS, resuspended in 1× binding buffer, and stained with PE‐Annexin V and 7‐AAD in the dark for 1 h. For stemness analysis, cells were labeled with FITC‐CD44 and PE‐CD24 antibodies (Invitrogen, USA) to determine the CD44^+^CD24^−^ population, and the ALDEFLUOR kit (STEMCELL, Canada) was used to assess ALDH^+^ cells. Stained cells were analyzed using a FACSCalibur flow cytometer (BD Biosciences, CA, USA).

For flow cytometry analysis of mouse subcutaneous tumors, tumor tissues were excised and transferred into 5 mL tubes, then finely minced with sterile scissors and washed twice with pre‐chilled PBS. Tumor digestion was performed using TESCA buffer containing collagenase type IV and DNase I at 37°C with gentle shaking for 2 h. The digested tissues were passed through a 50 µm nylon cell strainer to obtain single‐cell suspensions, followed by centrifugation at 1000 rpm for 5 min at 4°C. The supernatant was gently discarded, and the pellet was resuspended in three volumes of red blood cell lysis buffer, mixed gently, and incubated on ice for 2 min to remove erythrocytes. Cells were then washed twice with cold PBS and centrifuged at 500 g for 5 min at 4°C. To block Fc receptors, cells were incubated with anti‐mouse CD16/32 antibody (2 µL per sample) for 10 min on ice. After washing twice with cold PBS, the cell pellet was resuspended in 100 µL PBS and incubated with fluorochrome‐conjugated antibodies against indicated surface markers for 30 min at 4°C in the dark. After staining, cells were washed twice with 1 mL cold PBS, centrifuged (500 g, 5 min, 4°C), and resuspended in an appropriate volume of PBS for flow cytometric analysis.

### 2.13 ATP and Lactate Measurement

Cellular ATP levels were quantified using the ATP Assay Kit (Nanjing Jiancheng Bioengineering Institute, China) in 1 × 10^6^ transfected or treated cells. For lactate production, cells were incubated in serum‐free DMEM for 24 h, and lactate concentration was measured using a lactate assay kit (Nanjing Jiancheng Bioengineering Institute, China) according to the manufacturer's instructions.

### 2.14 Glycolysis Stress Test

The extracellular acidification rate (ECAR) was measured using the Seahorse XF Glycolysis Stress Test Kit (Agilent, USA). Transfected cells (2 × 10^4^) were seeded in XF 96‐well plates and incubated overnight. The medium was replaced with XF Base Medium (Agilent) and incubated for 1 h before the sequential addition of glucose (10 mM), oligomycin (1 µM), and 2‐deoxyglucose (2‐DG, 50 mM). Measurements were acquired using a Seahorse XFe96 Analyzer.

### 2.15 Sphere Formation Assay

Transfected or treated cells were seeded in Ultra‐Low Attachment 96‐well plates and cultured for 1 week in DMEM/F12 supplemented with 20 ng/mL human EGF, 20 ng/mL FGF, B27 (1:50), 5 µg/mL insulin, 100 U/mL penicillin, and 100 µg/mL streptomycin. Mammospheres were imaged under an optical microscope and sphere diameters were measured.

### 2.16 Patient‐Derived Organoid (PDO) Culture and Treatment

Fresh tumor tissues were minced and enzymatically digested at 37°C for 2 h using a cocktail containing 1% BSA, ITS‐G, Y‐27632 (5 µM), Primocin, HEPES (10 mM), hyaluronidase (1000 U/mL), and collagenases I and III. The digested mixture was filtered through a 100 µm strainer and centrifuged at 300× g for 5 min at 4°C. After red blood cell lysis using TAC buffer, cells were resuspended in growth factor‐reduced Matrigel (R&D Systems, USA), seeded in 48‐well plates, and incubated at 37°C for 1 h to solidify.

Organoid culture medium consisted of DMEM/F12 supplemented with 0.5 µg/mL R‐spondin‐1, 0.5 µg/mL Neuregulin 1, 5 ng/mL FGF7, 5 ng/mL EGF, 0.1 µg/mL Noggin, 20 ng/mL FGF10, 500 nM A83‐01, 5 µM Y‐27632, 500 nM SB202190, 1.25 mM N‐acetyl‐L‐cysteine, 5 mM nicotinamide, 1× Primocin, 1× GlutaMax, 1× HEPES, and 1× B27. Fresh medium was added every 3 days. Organoids were infected with either circZNF148‐overexpressing lentivirus or control virus 48 h post‐seeding. Organoid size and morphology were evaluated under a light microscope.

### 2.17 Proximity Ligation Assay (PLA)

The proximity ligation assay (PLA) was performed using the NaveniFlex Cell MR Kit (Navinci, Sweden) according to the manufacturer's protocol. Briefly, after fixation and permeabilization, cells were blocked at 37°C for 60 min in a humidified chamber. Primary antibodies were then applied and incubated overnight at 4°C. Following three washes with 1× TBS‐T, cells were incubated with Navenibody working solution at 37°C for 60 min, followed by TBS‐T washes at 37°C. Subsequently, Reaction 1 was added and incubated at 37°C for 30 min, with subsequent TBS‐T washing. Next, Reaction 2 was added and incubated at 37°C for 90 min, followed by washes. For nuclear staining, slides were incubated with DAPI for 10 min and imaged.

### 2.18 MTT Assay

A total of 1.5 × 10^3^ transfected or treated cells were seeded in 96‐well plates. At the indicated time points, 20 µL MTT solution (5 mg/mL) was added to each well and incubated for 4 h. The supernatant was discarded, 100 µL DMSO was added, and absorbance was measured at 490 nm (Bio‐Rad, USA) to generate growth curves.

### 2.19 Colony Formation Assay

Cells (1 × 10^3^) were seeded in 6 cm dishes and cultured for 10–14 days, with medium refreshed every 3 days. Colonies were fixed with methanol, stained with 0.5% crystal violet, photographed, and counted.

### 2.20 EdU Incorporation Assay

Cell proliferation was assessed using an EdU assay kit (RiboBio, China). Cells at 60%–70% confluence were incubated with 50 µM EdU for 2 h, fixed in 4% paraformaldehyde, permeabilized with 0.5% Triton X‐100, and stained with Apollo dye followed by Hoechst 33342. Images were captured using a fluorescence microscope.

### 2.21 Wound Healing Assay

Cells at 90% confluence in 24‐well plates were scratched using a sterile 10 µL pipette tip, rinsed with PBS, and cultured in serum‐free medium. Images were captured at 0 h and 48 h under a light microscope.

### 2.22 Transwell Assay

For migration assays, 6–8 × 10^4^ cells suspended in serum‐free DMEM were seeded into the upper chamber of a Transwell insert (8 µm pore; Corning, USA), while the lower chamber contained medium with 20% FBS. For invasion assays, the upper chambers were pre‐coated with Matrigel (BD Biosciences, USA). After 24–48 h, migrated/invaded cells were fixed with methanol, stained with crystal violet, and counted under a light microscope.

### 2.23 RNA Pulldown, Protein Visualization, and Mass Spectrometry

RNA pulldown assays were performed using the Pierce Magnetic RNA‐Protein Pull‐Down Kit (Thermo Fisher Scientific, USA) according to the manufacturer's instructions. Captured proteins were analyzed by western blot or visualized using Fast Silver Staining (Beyotime, China) or Coomassie Brilliant Blue staining (Solarbio, China). Mass spectrometry was conducted by Beijing Qinglian Biotech Co., Ltd.

### 2.24 RNA Immunoprecipitation (RIP) Assay

RIP assays were conducted using the Magna RIP Kit (Millipore, USA). Briefly, cell lysates were incubated with magnetic beads conjugated with specific antibodies overnight at 4°C. Co‐precipitated RNAs were extracted with TRIzol and analyzed by qRT‐PCR.

### 2.25 Immunohistochemistry (IHC)

Paraffin‐embedded tumor tissues from patients or mouse xenografts were sectioned at 4 µm, deparaffinized, and rehydrated through graded ethanol. After antigen retrieval and quenching of endogenous peroxidase activity, sections were incubated with primary antibodies overnight at 4°C, followed by HRP‐conjugated secondary antibodies. Visualization was achieved with DAB substrate, and nuclei were counterstained with hematoxylin. Images were acquired using a light microscope. Positive cell percentages and H‐scores were quantified using QuPath software. The staining intensity (*i*) was graded on a scale of 0 to 3: 0 (negative), 1 (weak), 2 (moderate), and 3 (strong). The percentage of cells at each intensity level (*Pi*) was evaluated from 0% to 100%. The H‐score was calculated using the formula: H‐score = Σ (*i* × *Pi*), which yields a theoretical range of 0 to 300. The final H‐score for each specimen was determined by averaging the values across three randomly selected representative regions.

### 2.26 In Vitro T Cell Co‐Culture Assay

Purified human CD8^+^ T cells were isolated from peripheral blood mononuclear cells (PBMCs) using the CD8^+^ T Cell Isolation Kit (Miltenyi Biotec, 130‐096‐495) according to the manufacturer's protocol. Briefly, PBMCs were incubated with a biotin‐conjugated antibody cocktail and anti‐biotin microbeads at 4°C, followed by magnetic separation using a MidiMACS Separator to obtain CD8^+^ T cells. CD8^+^ T cells were cultured at 1 × 10^6^ cells/mL in T cell expansion medium supplemented with recombinant human IL‐2 (10 ng/mL; PeproTech). On Day 0, cells were activated with ImmunoCult Human CD3/CD28 T Cell Activator (5 µg/mL final concentration) and incubated at 37°C in a humidified 5% CO_2_ atmosphere. Every 2–3 days, cultures were replenished with fresh medium containing IL‐2 (10 ng/mL) to maintain a cell density of 0.5–2 × 10^6^ cells/mL. After 7 days of expansion, CD8^+^ T cells were co‐cultured with adherent breast cancer cells (MDA‐MB‐231 or MDA‐MB‐468) at an effector‐to‐target (E:T) ratio of 3:1 in the continued presence of recombinant human IL‐2 (10 ng/mL) and low‐dose CD3/CD28 Activator (1 µg/mL). After 24 h of co‐culture, cells were collected for further analysis.

### 2.27 pH‐Clamped Experiments

To separate the effects of lactate from extracellular acidification, pH‐clamped experiments were performed. Both CD8^+^ T cells and TNBC cells were cultured under four controlled conditions: (1) standard medium (Control); (2) medium supplemented with 20 mM sodium lactate (NaLa); (3) an HCl‐acidified control; and (4) medium supplemented with 20 mM lactic acid (LA). The HCl‐acidified control was specifically pH‐matched to the LA group. Following treatment, CD8^+^ T cells were analyzed by flow cytometry for PD‑1 and TIM‑3 expression, and culture supernatants were collected for IFN‑γ and TNF‑α detection via ELISA. Additionally, whole‑cell lysates of TNBC cells were subjected to western blotting to evaluate global and PD‑L1‑specific lactylation levels.

### 2.28 Animal Experiments

For the durvalumab treatment experiments, human peripheral blood mononuclear cells (PBMCs) isolated from healthy donors were intravenously injected into SCID mice (1.0 × 10^7^ cells per mouse) to establish Hu‐PBL‐SCID mice. MDA‐MB‐231 cells (1 × 10^6^ cells/mouse) stably expressing pLCDH‐ciR or circZNF148 were subcutaneously implanted. When tumors became palpable, mice were randomly allocated into three treatment groups: isotype IgG control (2 mg/kg), durvalumab monotherapy (2 mg/kg), or durvalumab (2 mg/kg) combined with 2‐DG (100 mg/kg), resulting in six experimental groups (n = 5 per group). Treatments were administered intraperitoneally every 3 days for a total of six administrations. Tumor volumes were measured every 5 days and calculated using the formula (length × width^2^)/2. At the study termination, tumors were excised and tumor weight was measured. The tissues were sectioned for hematoxylin and eosin (H&E) staining, immunohistochemical (IHC), ISH, and TUNEL analyses. For pulmonary metastasis assay, MDA‐MB‐231 cells (5 × 10^5^ cells) with or without circZNF148 overexpression were injected into the lateral tail veins of 4‐ to 6‐week‐old BALB/c nude female mice (n = 5 per group). At the endpoint, the lungs were harvested and the metastatic nodules were counted. All of the experimental procedures involving animals were approved by Shandong University Animal Care and Use Committee.

### 2.29 Statistical Analysis

Statistical analysis was performed using GraphPad Prism 10.3.1 and R software (version 4.3.3). Data are presented as mean ± standard deviation (SD) from at least three independent biological replicates. Comparisons between two groups or among multiple groups were assessed using Student's t‐test or one‐way ANOVA, respectively. The Chi‐square test was used to analyze associations between circZNF148 expression and categorical clinicopathological variables. For clinical cohort analysis, receiver operating characteristic (ROC) curves were generated using overall survival (OS) status as the clinical endpoint. The optimal cut‐off value for continuous circZNF148 expression was determined by calculating the maximum Youden index (Sensitivity + Specificity – 1). To mitigate the risk of overfitting and confirm the robustness of the selected threshold, internal validation was performed using 2000 stratified bootstrap resamples to estimate the 95% confidence interval (CI) of the AUC. Kaplan‐Meier curves were generated and compared using the log‐rank test. Univariate and multivariate Cox proportional hazards regression models were used to identify independent prognostic factors. A *p*‐value < 0.05 was considered statistically significant.

## 3 Results

### 3.1 Identification of circZNF148 as a Glucose Metabolism‐Related circRNA in Breast Cancer

Previous studies have reported that breast cancer cells with higher metastatic potential exhibited enhanced glycolytic activity [20, 21]. In our study, we examined a constructed subline derived from MDA‐MB‐231 cells with enhanced lung metastatic capacity and found significantly elevated levels of glucose‐6‐phosphate (G6P), increased ATP production, and higher lactate accumulation compared to the parental cells, further supporting the association between glucose metabolism and metastatic potential (Figure S1A). To identify circRNAs associated with both enhanced glycolysis and lung metastasis potential, we conducted a comparative expression analysis across two experimental models (Figure 1A). First, we profiled circRNA expression in two cell lines subjected to low‐glucose conditions for 24 h relative to their normal counterparts. Importantly, both culture pH and cell viability under low‑glucose treatment were comparable to those under normal‑glucose conditions (Figure S1B,C). CircRNAs exhibiting an average expression level (Aver exp) > 5 and a fold change > 4 were considered significantly upregulated, yielding 40 candidate circRNAs. Concurrently, we analyzed circRNA expression in a lung metastasis model by comparing highly metastatic cells to their parental counterparts. Applying the same criteria, we identified 614 upregulated circRNAs. Intersection of these two datasets revealed three commonly upregulated circRNAs (Figure 1B). Among them, hsa_circ_0121742, hereafter referred to as circZNF148, was selected for further investigation due to its most pronounced upregulation in both glycolysis‐enhanced and metastasis‐prone cellular models, as validated by qRT‐PCR and FISH assays (Figure 1C,D, Figure S1D,E). circZNF148 is generated from exons 4 and 5 of the *ZNF148* gene located on chromosome 3q24 (chr3:125 006 946–125 032 500). Its biogenesis involves back‐splicing, wherein the 5′ splice donor site of exon 5 joins covalently to the 3′ splice acceptor site of exon 4, generating a covalently closed circular RNA with a length of 475 nucleotides (Figure 1E). Sanger sequencing confirmed the head‐to‐tail splicing junction, validating the circular structure of circZNF148 (Figure 1E).

**FIGURE 1:**
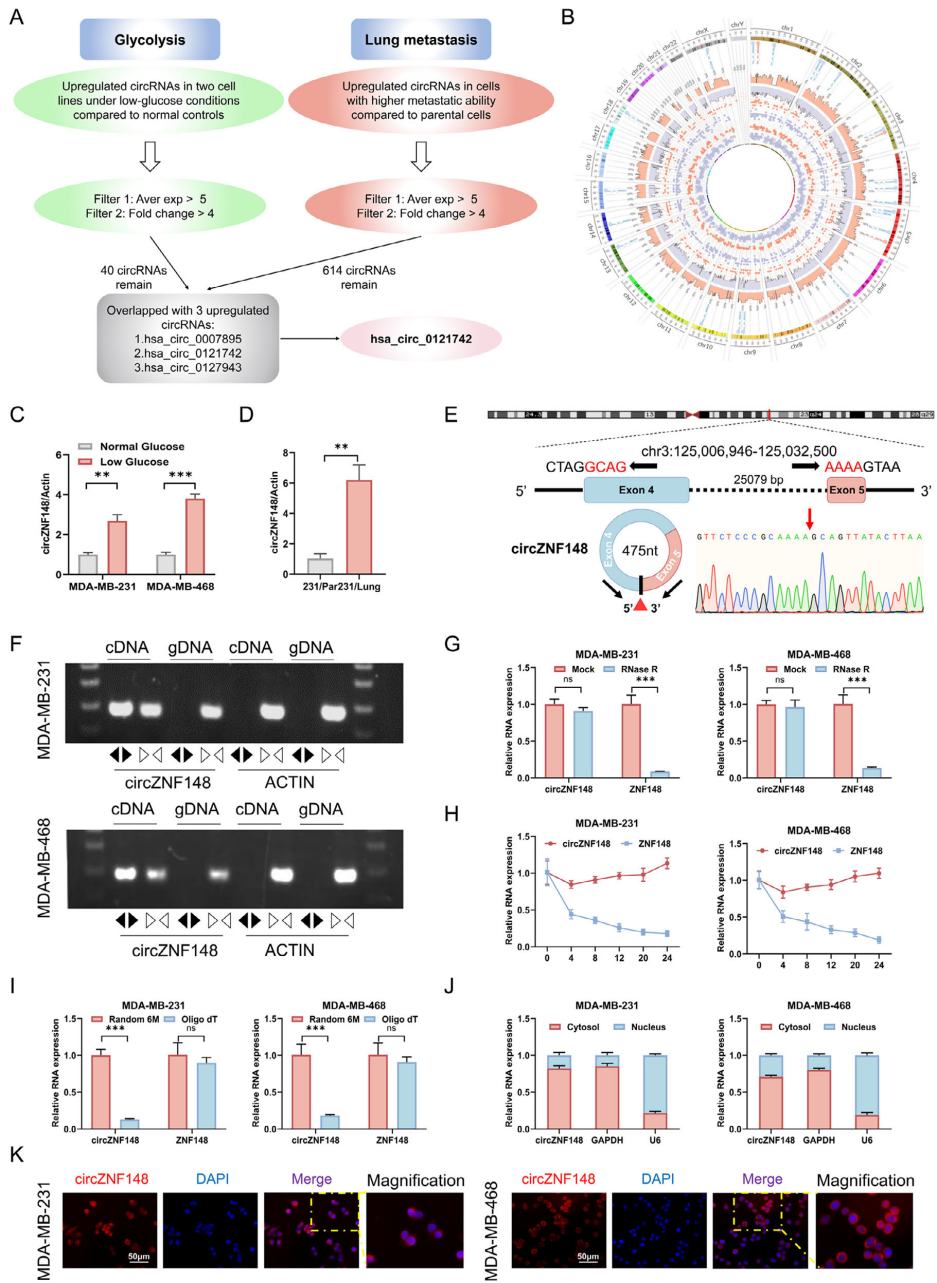
Identification and characterization of glycolysis‐ and lung metastasis‐associated circZNF148 in breast cancer cells. (A) Schematic of the experimental strategy to identify circRNAs linked to enhanced glycolysis under metabolic stress and lung metastatic potential in breast cancer. Diagram highlights three overlapping circRNAs (average expression > 5, fold change > 4) upregulated in low‐glucose‐stimulated (1 g/L glucose for 24 h) and highly metastatic cell populations. (B) Circos plot visualization of differentially expressed circRNAs, with the three overlapping candidates (red) showing significant upregulation. (C,D) qRT‐PCR validation of hsa_circ_0121742 expression in low‐glucose‐treated (24 h, C) and lung metastasis‐prone (D) cell models. (E) Genomic structure of hsa_circ_0121742, derived from exons 4 and 5 of the *ZNF148* gene (chr3:125 006 946–125 032 500), with back‐splicing forming a 475‐nt circular RNA. Sanger sequencing confirmed the head‐to‐tail junction (red arrow). (F) PCR amplification using convergent and divergent primers confirmed the circular nature of circZNF148 (detected only from cDNA, not gDNA). (G) RNase R resistance assay demonstrated the stability of circZNF148 compared to linear *ZNF148* mRNA. (H) Actinomycin D (ActD) chase assay revealed the enhanced RNA stability of circZNF148 relative to linear *ZNF148*. (I) qRT‐PCR with oligo dT or random primers confirmed the absence of a poly(A) tail in circZNF148. (J) Subcellular fractionation analysis confirmed the predominant cytoplasmic localization of circZNF148. U6 and GAPDH served as nuclear and cytoplasmic loading controls, respectively. (K) Fluorescence in situ hybridization (FISH) further validated cytoplasmic enrichment of circZNF148, using a Cy3‐labeled probe (red). Nuclei were counterstained with DAPI (blue). Scale bars, 50 µm.

To further confirm its circular nature, we employed both convergent and divergent PCR primers. As expected, divergent primers amplified circZNF148 from cDNA but not from genomic DNA, whereas convergent primers detected the linear transcript (Figure 1F). Moreover, circZNF148 exhibited marked resistance to RNase R, a characteristic exonuclease that degrades linear RNAs, while its linear counterpart *ZNF148* was rapidly degraded (Figure 1G). Consistent with increased stability, actinomycin D (ActD) treatment revealed a significantly longer half‐life for circZNF148 compared to linear *ZNF148* mRNA (Figure 1H). Additionally, circZNF148 was barely detectable when reverse transcription was performed using oligo dT primers, in contrast to random hexamer primers, indicating the absence of a 3’ poly(A) tail (Figure 1I). Subcellular fractionation assays and fluorescence in situ hybridization (FISH) assays demonstrated that circZNF148 is predominantly localized in the cytoplasm (Figure 1J,K). Furthermore, circZNF148 expression was significantly elevated in breast cancer cell lines compared to normal mammary epithelial cells (Figure S1F), suggesting a potential oncogenic role in breast cancer progression.

### 3.2 CircZNF148 Promotes Glycolysis and Metastasis in Triple‐Negative Breast Cancer

To delineate the functional role of circZNF148 in TNBC, we first transfected specific siRNAs targeting its back‐splicing junction into MDA‐MB‐231 and MDA‐MB‐468 cells, which can efficiently knock down the expression of circZNF148 without affecting the mRNA levels of its linear host gene, *ZNF148* (Figure S2A,B). Our results indicated that circZNF148 knockdown significantly impaired cell proliferation, DNA replication, and colony formation capacities, while increasing apoptotic rates in both cell lines (Figure S2C‐F). Moreover, the migration and invasion abilities were attenuated after circZNF148 knockdown in MDA‐MB‐231 and MDA‐MB‐468 cells (Figure S3A,B), which was further validated in another TNBC cell line, BT‐549 (Figure S3C,D), suggesting its critical role in regulating aggressive phenotypes. Conversely, ectopic overexpression of circZNF148 enhanced proliferative, migratory, and invasive capabilities while suppressing apoptosis (Figures S4 and S5), further supporting its oncogenic function in TNBC.

The effect of circZNF148 on modulating glycolytic activity and the link between metabolic reprogramming and tumor progression were further evaluated. ECAR analysis using the Seahorse XF Analyzer revealed that circZNF148 knockdown significantly reduced glycolytic flux in breast cancer cells (Figure 2A). This was corroborated by reduced intracellular levels of lactate, ATP, and glucose‐6‐phosphate (G6P) upon circZNF148 knockdown (Figure 2B–D, Figure S6A). Additionally, circZNF148 knockdown impaired 3D spheroid formation (Figure S6B), a functional hallmark of tumor‐initiating potential. Importantly, limiting dilution assays (LDA) in vitro showed that circZNF148‐knockdown cells exhibited a markedly reduced tumor‐initiating frequency compared to control cells (Figure S6C), highlighting its essential role in maintaining CSC functionality. Flow cytometry analysis further demonstrated a significant reduction in the proportion of ALDH+ cells and CD44^+^CD24^−^ cancer stem‐like cells upon circZNF148 knockdown (Figure S6D), indicating attenuated tumor stemness. Conversely, circZNF148 overexpression exerted the opposite effect, leading to enhanced glycolytic activity and tumor stemness (Figure S7). Consistently, key glycolytic enzymes, epithelial‐mesenchymal transition (EMT) and stemness markers, and associated signaling pathways were downregulated upon circZNF148 silencing and upregulated upon its overexpression (Figure S8). These findings indicate that circZNF148 enhances glycolytic flux and sustains aggressive phenotypes in TNBC. Subsequently, we investigated whether circZNF148 exerts its oncogenic effects via regulation of glycolysis. To test this, we performed functional rescue experiments using the glycolysis inhibitors 2‐deoxy‐D‐glucose (2‐DG, hexokinase inhibitor) and oxamate (LDHA inhibitor). Importantly, no significant difference in extracellular pH was observed between control and inhibitor‑treated groups (Figure S9A). As expected, circZNF148 overexpression promoted migration, invasion, and stemness in TNBC cells, however, these pro‐tumorigenic effects were significantly reversed upon treatment with either inhibitor (Figure 2E,F, Figure S9B–D). Notably, we extended these findings to a more physiologically relevant model by establishing patient‐derived organoids. In 3D organoid cultures, circZNF148 overexpression led to increased organoid size, while treatment with 2‐DG or oxamate markedly suppressed organoid growth (Figure 2G, Figure S9E). Collectively, these results suggest that circZNF148 promotes tumor aggressiveness in a glycolysis‐dependent manner.

**FIGURE 2:**
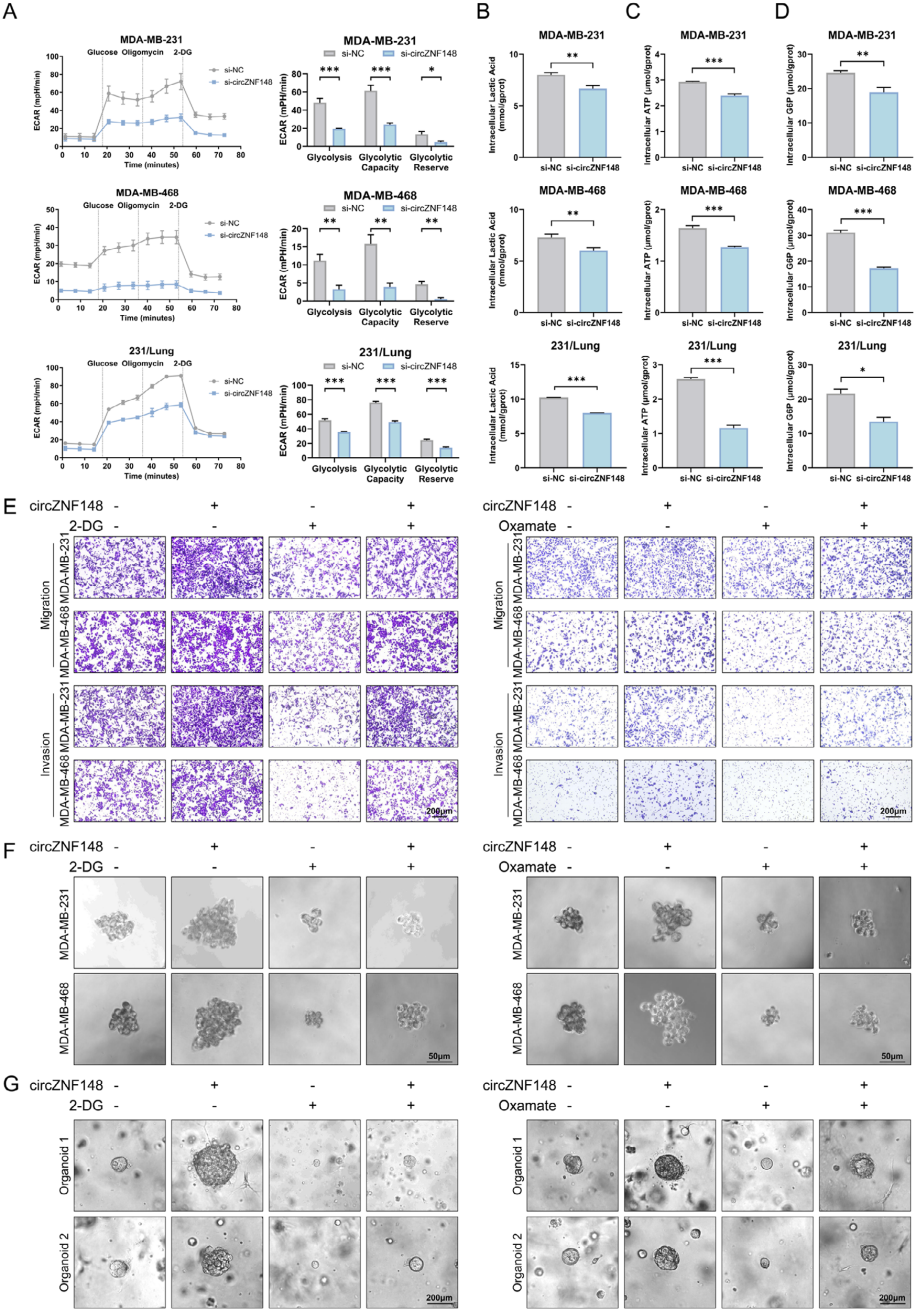
circZNF148 drives glycolytic reprogramming, metastatic progression, and stemness in TNBC. (A) Seahorse extracellular acidification rate (ECAR) analysis of circZNF148‐knockdown MDA‐MB‐231, MDA‐MB‐468 and lung‐metastatic subline (231/Lung). Quantification of glycolytic parameters is shown (right). (B‐D) Intracellular lactate, ATP, and glucose‐6‐phosphate (G6P) levels in circZNF148‐knockdown cells. (E) Transwell migration and invasion assays were performed in control cells treated with glycolysis inhibitors (2‐DG, oxamate), with partial rescue by circZNF148 overexpression. Scale bars, 200 µm. (F) Spheroid assays were performed in control cells treated with glycolysis inhibitors (2‐DG, oxamate), with partial rescue by circZNF148 overexpression. Scale bars, 50 µm. (G) Patient‐derived organoid (PDO) models with circZNF148 overexpression exhibit altered responses to glycolysis inhibition. Scale bars, 200 µm. *p < 0.05; **p < 0.01; ***p < 0.001.

### 3.3 IGF2BP2 Facilitates the Biogenesis of circZNF148 in Triple‐Negative Breast Cancer

Having established circZNF148 as an oncogenic circRNA involved in TNBC progression, we next sought to elucidate the upstream regulatory mechanisms underlying its high expression. Previous studies have demonstrated that specific RNA‐binding proteins (RBPs) can bind to the flanking sequences of circRNAs to modulate their biogenesis [22]. Utilizing the RBPmap and ATtRACT databases, we predicted potential RBPs that might interact with the flanking regions of circZNF148, and the intersection of these two databases yielded 37 candidate RBPs. We further cross‐referenced these candidates with upregulated genes in TNBC versus non‐TNBC tissues using the TCGA‐BRCA dataset, identifying three candidates: IGF2BP2, KHDRBS3, and YBX1 (Figure S10A). To verify the effects of these candidates on circZNF148 expression, specific siRNAs and overexpression vectors were transfected into TNBC cells. Our results showed that the expression of IGF2BP2, but not KHDRBS3 or YBX1, significantly affected circZNF148 levels (Figure S10B,C). Specifically, IGF2BP2 overexpression markedly increased circZNF148 abundance, whereas its knockdown inhibited circZNF148 expression. Notably, the mRNA levels of the linear host gene *ZNF148* remained relatively stable, indicating that IGF2BP2 drives post‐transcriptional cyclization rather than host gene transcription. Furthermore, RNA immunoprecipitation (RIP) assay demonstrated that IGF2BP2 could bind to both the upstream and downstream flanking intron sequences of the *ZNF148* pre‐mRNA, indicating that IGF2BP2 facilitates the biogenesis of circZNF148 via back‐splicing (Figure S10D). Given that circZNF148 was identified as a glucose‐responsive circRNA, we further evaluated whether IGF2BP2 also responds to metabolic stress. We found that the expression of IGF2BP2 was significantly upregulated under low‐glucose conditions, mirroring the expression pattern of circZNF148 (Figure S10E,F). Moreover, IGF2BP2 knockdown significantly attenuated the induction of circZNF148 triggered by low glucose treatment, suggesting that IGF2BP2 serves as a key mediator of circZNF148's response to metabolic stress (Figure S10G). To exclude the possibility that IGF2BP2 elevates circZNF148 levels via enhanced RNA stability, we measured the degradation rate of circZNF148 after knocking down IGF2BP2 in cells exposed to actinomycin D. The results showed that loss of IGF2BP2 did not markedly affect the degradation of circZNF148 (Figure S10H), further supporting that IGF2BP2 upregulates circZNF148 expression by promoting its cyclization. Collectively, these findings demonstrate that IGF2BP2 acts as an upstream regulator that binds to the flanking introns of the *ZNF148* pre‐mRNA to facilitate circZNF148 biogenesis and maintain its high expression in TNBC, particularly under conditions of metabolic reprogramming.

### 3.4 circZNF148 Promotes Metastasis Through HK1‐Dependent Enhancement of Glycolysis

To explore potential molecular effectors of circZNF148, RNA pull‐down assays followed by silver staining and mass spectrometry were conducted (Figure S11A). The unbiased screen identified several glycolysis‐related enzymes as potential binding partners, with hexokinase 1 (HK1) emerging as a top candidate (Figure S11B,C). The interaction between circZNF148 and HK1 was validated by RIP and RNA pull‐down assays (Figure 3A, Figure S11D), which was further confirmed by combined fluorescence in situ hybridization (FISH) and immunofluorescence (IF) analysis, revealing the cytoplasmic colocalization of circZNF148 and HK1 (Figure 3B). Moreover, both circZNF148 and HK1 were upregulated under low‐glucose conditions (Figure 3B), suggesting a coordinated adaptive response to metabolic stress. To map the interaction regions between circZNF148 and HK1, three truncated circZNF148 constructs based on structure prediction were generated (Figure 3C, Figure S11E). RNA pull‐down assay revealed that all three circZNF148 fragments were capable of interacting with HK1 (Figure 3C, Figure S11F). Additionally, HK1 contains two functional domains: an N‐terminal regulatory domain that modulates enzymatic activity and a C‐terminal catalytic domain responsible for glucose phosphorylation through glucose binding and ATP hydrolysis [23]. To further define the interaction interface, we generated two HK1 truncations encompassing the N‐terminal and C‐terminal domains, respectively. RIP assay demonstrated that the N‐terminal regulatory domain of HK1 was primarily responsible for binding circZNF148 (Figure 3D). These findings suggest that circZNF148 engages the N‐terminal region of HK1 through a broad interaction.

**FIGURE 3:**
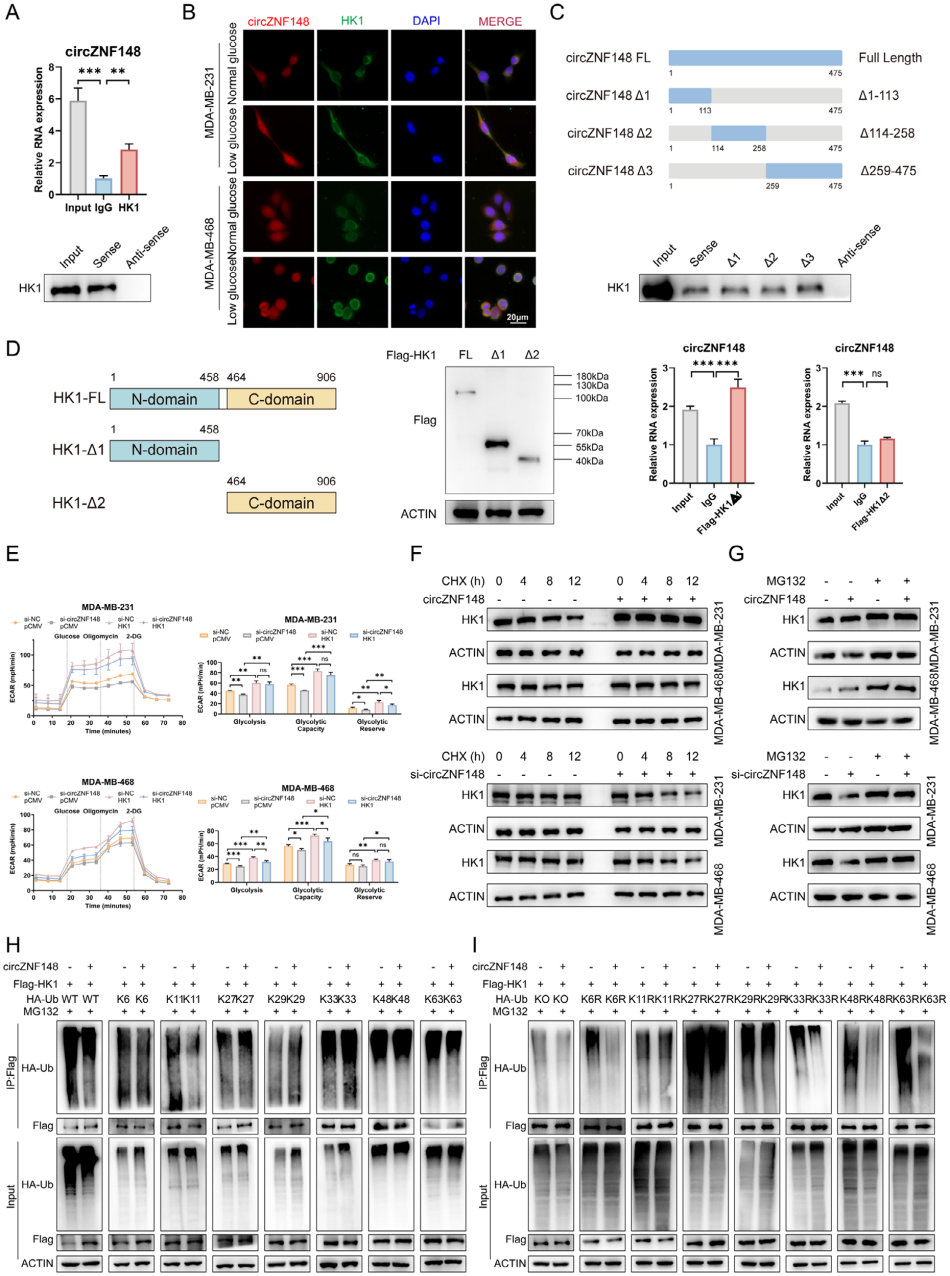
circZNF148 stabilizes HK1 by preventing its K11‐linked ubiquitination and degradation. (A) RIP and RNA pull‐down assays confirm the endogenous circZNF148‐HK1 interaction. (B) Combined FISH (circZNF148, red) and IF (HK1, green) show cytoplasmic colocalization. Cells were cultured under normal (4.5 g/L glucose) or low‐glucose (1 g/L glucose) conditions for 24 h prior to fixation. Scale bars, 20 µm. (C) Schematic of truncated circZNF148 (Δ1‐Δ3), followed by RNA pull‐down. (D) Schematic of truncated HK1, and western blot confirms the expression and molecular weight. RIP assay maps the N‐terminal domain of HK1 binding to circZNF148. (E) ECAR assays demonstrating that the glycolytic defect upon circZNF148 loss is rescued by HK1 overexpression in cancer cells. Quantification of glycolytic parameters is shown. (F) Following circZNF148 overexpression or knockdown, and treatment with 100 µg/mL CHX for indicated time, breast cancer cell lysates were subjected to western blot. Protein levels were quantified by ImageJ software and normalized to β‐actin. (G) Following circZNF148 overexpression or knockdown, and treatment with MG132 (20 µM) for 5 h, breast cancer cell lysates were subjected to western blot. Protein levels were quantified by ImageJ software and normalized to β‐actin. (H) HEK293T cells were transfected with Flag‐HK1 and vector/circZNF148 together with wild‐type (WT) ubiquitin or different ubiquitin mutants. After treatment with MG132 (20 µM) for 5 h, the cells were collected for co‐immunoprecipitation (co‐IP) and western blot assays. (I) HEK293T cells were transfected with Flag‐HK1 and vector/circZNF148 together with K‐to‐R ubiquitin mutants. After treatment with MG132 (20 µM) for 5 h, the cells were collected for co‐IP and western blot assays. ns, no significance; *p < 0.05; **p < 0.01; ***p < 0.001.

Rescue experiments were further performed to determine whether HK1 mediates the functional effects of circZNF148. The results indicated that circZNF148 knockdown significantly impaired cell migration, reduced stem‐like properties, and suppressed glycolytic metabolism, as shown by reduced ECAR and lower levels of intracellular glucose, G6P, ATP, and lactate (Figure 3E, Figure S12). Notably, ectopic expression of HK1 effectively reversed these phenotypic and metabolic alterations (Figure 3E, Figure S12). These results demonstrate that HK1 is a key downstream effector of circZNF148 in promoting cancer cell migration, stemness, and glycolytic activity.

### 3.5 circZNF148 Stabilizes HK1 by Suppressing K11‐Linked Ubiquitination

Our findings demonstrated that circZNF148 positively correlates with HK1 protein expression in TNBC cells, while no corresponding changes in *HK1* mRNA levels were observed upon either overexpression or knockdown of circZNF148 (Figure S13A,B). This dissociation between protein and transcript levels suggests that circZNF148 might regulate HK1 through post‐translational mechanisms, rather than transcriptional or mRNA stability control. We first assessed the impact of circZNF148 on HK1 protein stability. Treatment of TNBC cells with cycloheximide (CHX), a potent inhibitor of de novo protein synthesis, revealed that circZNF148 overexpression significantly extended the half‐life of HK1, while knockdown of circZNF148 accelerated HK1 degradation, supporting its role in maintaining HK1 abundance (Figure 3F, Figure S13C,D). Furthermore, MG132, a proteasome inhibitor, led to HK1 accumulation in control cells (Figure 3G), suggesting that HK1 is primarily degraded via the ubiquitin‐proteasome system. Strikingly, the effect of circZNF148 knockdown on HK1 reduction was reversed by MG132, demonstrating that circZNF148 protects HK1 from proteasome‐dependent degradation (Figure 3G, Figure S13E).

To determine whether circZNF148‐mediated stabilization of HK1 was attributable to the suppression of ubiquitin‐mediated degradation, we co‐transfected TNBC cells with HA‐tagged ubiquitin (HA‐Ub) and either circZNF148 overexpression or control vectors. Immunoprecipitation of HK1 followed by anti‐HA immunoblotting showed that circZNF148 overexpression markedly diminished the overall ubiquitination of HK1 (Figure 3H, Figure S13F). Given that distinct polyubiquitin chain linkages dictate distinct functional outcomes, we further analyzed the linkage specificity. Notably, circZNF148 overexpression selectively suppressed K11‐linked ubiquitination of HK1, with no significant effect on other lysine linkages (K6, K27, K29, K33, K48, K63) (Figure 3H). Furthermore, we employed a panel of ubiquitin mutants in which each lysine residue was individually substituted with arginine (K‐to‐R). Consistent with the linkage‐specific effects, expression of K11R ubiquitin abolished HK1 polyubiquitination, whereas other K‐to‐R mutants retained detectable ubiquitin conjugation (Figure 3I). Moreover, circZNF148 overexpression strongly inhibited HK1 ubiquitination mediated by wild‐type and all mutant ubiquitins, except K11R, where no further reduction was observed (Figure 3I), indicating that circZNF148 specifically interferes with K11‐linked chain formation of HK1. Collectively, these results demonstrate that circZNF148 stabilizes HK1 by selectively inhibiting K11‐linked polyubiquitination, a chain topology classically associated with proteasomal targeting, thereby preventing its degradation.

### 3.6 circZNF148 Acts as a Molecular Scaffold to Facilitate the HK1‐ADRM1 Interaction and Block HK1 Degradation

Having established that circZNF148 suppresses K11‐linked ubiquitination of HK1, we next sought to identify the molecular machinery through which this regulation occurs. We analyzed the proteomic profile of circZNF148‐interacting proteins from RNA pull‐down assays, and identified the Adhesion Regulating Molecule 1 (ADRM1) as a candidate circZNF148‐interacting protein (Figure S14A). ADRM1 is a well‐characterized ubiquitin receptor of the 19S proteasomal subunit, responsible for recognizing ubiquitinated substrates and recruiting deubiquitinating enzymes [24]. RIP and RNA pull‐down assays confirmed the binding between circZNF148 and ADRM1 in TNBC cells (Figure S14B,C), suggesting a potential role in bridging HK1 to the ubiquitin regulatory machinery. To explore the structural basis of this interaction, we performed molecular docking using HDock, which revealed a high‐affinity binding interface between ADRM1 and HK1 (PDBePISA score: ‐272.10) (Figure 4A). Consistent with this prediction, co‐IP and IF assays demonstrated **a** robust interaction and intracellular co‐localization between ADRM1 and HK1 (Figure S14D–F). Truncation‐based co‐IP mapping further revealed that the N‐terminal regulatory domain of HK1 is responsible for ADRM1 binding (Figure 4B). To further define the precise structural architecture of this coordinated assembly, we generated three ADRM1 truncation mutants (N‐terminal, middle, and C‐terminal). Co‐IP, RIP, and RNA pull‐down analyses collectively revealed a precise modular binding pattern: the N‐terminal regulatory domain of HK1 interacts with the N‐terminal domain of ADRM1 (Figure 4B, Figure S14G,H), while circZNF148 binds to its middle domain, with all three of its fragments showing binding affinity (Figure S14C,I). Crucially, circZNF148 overexpression enhanced the HK1‐ADRM1 interaction, as evidenced by increased co‐IP efficiency in both endogenous and exogenous settings (Figure 4C, Figure S15A) and enhanced IF co‐localization (Figure S15B). To further validate the binding enhancement at the single‐cell level, proximity ligation assay (PLA) was performed, showing that circZNF148 overexpression markedly increased the number of HK1‐ADRM1 interaction foci (Figure 4D). Conversely, circZNF148 knockdown reduced the formation of HK1‐ADRM1 complex (Figure 4D, Figure S15C–E). These findings prompted us to investigate whether circZNF148 and ADRM1 operate in the same pathway.

**FIGURE 4:**
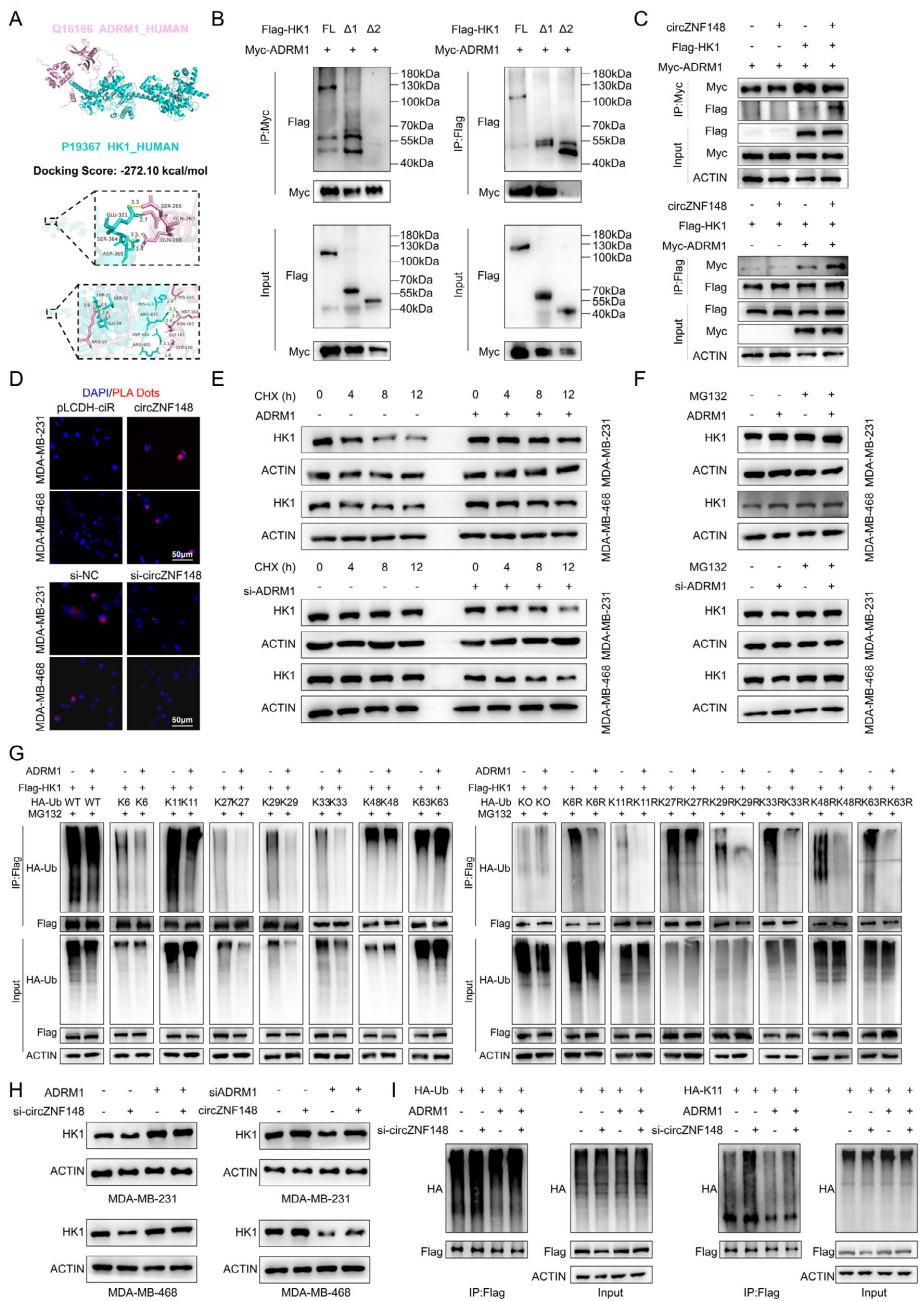
circZNF148 stabilizes HK1 via ADRM1‐mediated protection from K11‐linked ubiquitination. (A) Molecular docking (HDock) and PDBePISA scoring (score: −272.10) visualized in PyMOL, showing complementarity between HK1 (blue) and ADRM1 (red). (B) Truncation‐based Co‐IP mapping HK1‐ADRM1 interaction to HK1 N‐terminal domain. (C) Co‐IP confirming that circZNF148 overexpression elevates HK1‐ADRM1 interaction. (D) PLA confirming that circZNF148 overexpression elevates HK1‐ADRM1 interaction foci. Scale bars, 50 µm. (E) Following ADRM1 overexpression or knockdown, and treatment with 100 µg/mL CHX for indicated time, breast cancer cell lysates were subjected to western blot. Protein levels were quantified by ImageJ software and normalized to β‐actin. (F) Following ADRM1 overexpression or knockdown, and treatment with MG132 (20 µM) for 5 h, breast cancer cell lysates were subjected to western blot. (G) HEK293T cells were transfected with Flag‐HK1 and vector/Myc‐ADRM1 together with WT ubiquitin or different ubiquitin mutants. After treatment with MG132 (20 µM) for 5 h, the cells were collected for co‐IP and western blot assays. (H) Western blot analysis showing that ADRM1 overexpression rescues HK1 levels upon circZNF148 knockdown, and circZNF148 overexpression rescues HK1 levels upon ADRM1 knockdown. (I) Ubiquitination rescue assays show that overexpression of ADRM1 suppresses the enhanced HK1 ubiquitination (total and K11‐linked) induced by circZNF148 knockdown.

As expected, the half‐life of HK1 was markedly prolonged or shortened upon ADRM1 overexpression or knockdown (Figure 4E, Figure S15F), as determined by CHX chase assays. Moreover, MG132 treatment did not further increase HK1 stability in ADRM1‐overexpressing cells but effectively restored HK1 protein levels in ADRM1‐knockdown cells (Figure 4F), indicating that ADRM1 protects HK1 from degradation primarily through the ubiquitin‐proteasome system. Consistently, ADRM1 overexpression specifically reduced K11‐linked ubiquitination of HK1 without affecting other major ubiquitin chain types (Figure 4G), recapitulating the stabilizing effects observed upon circZNF148 overexpression. To determine whether ADRM1 functions downstream of circZNF148 in regulating HK1 stability, we performed rescue experiments. Overexpression of ADRM1 restored HK1 protein levels in circZNF148‐knockdown cells, whereas ADRM1 knockdown abolished the upregulation of HK1 induced by circZNF148 overexpression (Figure 4H), indicating that **ADRM1 is essential for circZNF148‐mediated HK1 stabilization**. Additionally, ADRM1 overexpression reduced overall and K11‐linked ubiquitination of HK1 induced by circZNF148 knockdown (Figure 4I). These findings collectively indicate that ADRM1 is a key effector in the circZNF148‐driven pathway that suppresses K11‐linked ubiquitination and subsequent proteasomal degradation of HK1.

Previous studies revealed that proteasomal ubiquitin receptor ADRM1 could recruit the deubiquitinase UCH37 to mediate substrate‐specific deubiquitination [25]. Given the suppressive **effect** of circZNF148 on K11‐linked ubiquitination of HK1, we hypothesized that it may facilitate the formation of a deubiquitination complex involving ADRM1 and UCH37. To test this, we first confirmed the interaction between ADRM1 and UCH37 in TNBC cells by co‐IP and IF assays (Figure 5A,B, Figure S16A). RNA pull‐down assays using biotin‐labeled circZNF148 revealed robust enrichment of ADRM1, but no detectable binding to UCH37 (Figure S14C), indicating that circZNF148 specifically interacts with ADRM1 but not with UCH37 directly. Nevertheless, co‐IP and IF experiments showed that UCH37 physically associates with HK1, and this interaction was enhanced upon circZNF148 overexpression (Figure 5C,D, Figure S16B–D), suggesting that UCH37 is recruited to HK1 in a circZNF148‐dependent manner, likely through an ADRM1‐mediated bridge. Importantly, co‐IP assays demonstrated that UCH37 binds to the C‐terminal domain of ADRM1 (Figure S16E). Together with the above domain‑mapping data showing that HK1 and circZNF148 bind the N‐terminal and middle domains of ADRM1, respectively (Figure 4B, Figure S14C,G–I), these findings indicate that all three components occupy distinct, non‐overlapping regions on ADRM1. This spatial separation avoids steric hindrance and allows for stable multimeric assembly. We further performed 3D molecular docking to visualize the spatial arrangement of this complex (Figure S16F). The docking model illustrates that circZNF148 acts as a molecular clamp bridging ADRM1 and HK1. Crucially, consistent with our biochemical mapping, this clamped conformation does not sterically occlude the UCH37‐binding region on ADRM1, thereby physically accommodating the recruitment of UCH37. Importantly, neither the protein nor mRNA levels of ADRM1 or UCH37 were altered by circZNF148 overexpression or knockdown (Figure S17), indicating that circZNF148 regulates their functional engagement, rather than expression. Significantly, UCH37 overexpression prolonged the half‐life of HK1 protein, whereas UCH37 knockdown accelerated HK1 degradation (Figure 5E), phenocopying the effects observed upon circZNF148 depletion. Moreover, UCH37 overexpression increased HK1 protein levels, while its knockdown led to decreased expression of HK1 (Figure 5F). These changes were markedly attenuated in the presence of MG132 (Figure 5F), indicating that UCH37 stabilizes HK1 by suppressing its proteasomal degradation. To determine whether UCH37 is functionally required for circZNF148‐mediated HK1 stabilization, we performed a series of rescue experiments. Notably, overexpression of UCH37 rescued HK1 protein levels in circZNF148‐knockdown cells, while UCH37 knockdown abrogated the stabilizing effect of circZNF148 overexpression on HK1 (Figure 5G). Furthermore, UCH37 overexpression reduced the K11‐linked ubiquitination of HK1 induced by circZNF148 knockdown (Figure 5H). These results demonstrate that UCH37 is essential for circZNF148‐mediated HK1 deubiquitination and accumulation.

**FIGURE 5:**
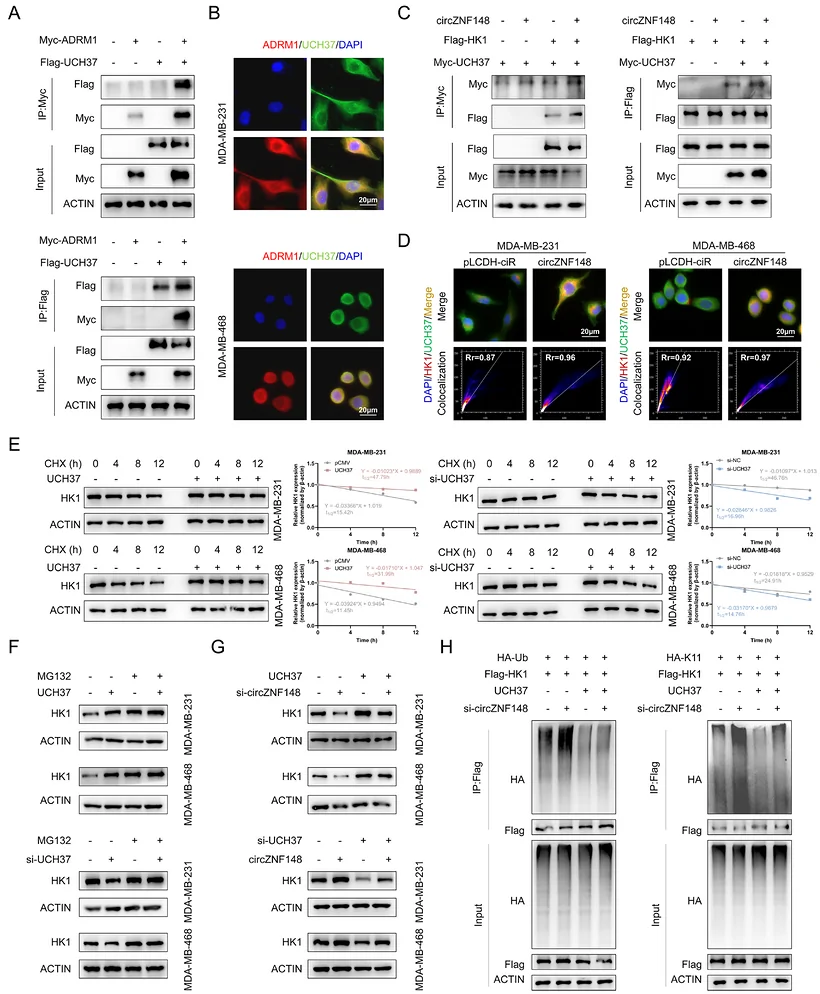
circZNF148 scaffolds an ADRM1‐UCH37 complex to deubiquitinate and stabilize HK1. (A) Co‐IP assays in HEK293T cells confirm the exogenous interaction between ADRM1 and UCH37. (B) IF staining demonstrates the co‐localization of ADRM1 and UCH37 in the cytoplasm. Scale bars, 20 µm. (C) Co‐IP assays show that UCH37‐HK1 interaction is strengthened upon circZNF148 overexpression. (D) IF staining reveals enhanced recruitment of UCH37 to HK1 upon circZNF148 overexpression. Scale bars, 20 µm. (E) Following UCH37 overexpression or knockdown, and treatment with 100 µg/mL CHX for indicated time, breast cancer cell lysates were subjected to western blot. Protein levels were quantified by ImageJ software and normalized to β‐actin. (F) Following UCH37 overexpression or knockdown, and treatment with MG132 (20 µM) for 5 h, breast cancer cell lysates were subjected to western blot. (G) Rescue experiments show that UCH37 restores HK1 protein levels diminished by circZNF148 knockdown, while UCH37 knockdown abolishes the HK1‐stabilizing effect of circZNF148 overexpression. (H) Ubiquitination rescue assays show that overexpression of UCH37 suppresses the enhanced HK1 ubiquitination (total and K11‐linked) induced by circZNF148 knockdown.

We next extended these findings to functional phenotypes relevant to tumor aggressiveness. circZNF148 knockdown suppressed metastatic ability, reduced tumor stemness, and impaired glycolytic flux, while co‐overexpression of ADRM1 rescued these defects in circZNF148‐deficient cells (Figure S18A–C). Similarly, overexpression of UCH37 also reversed the phenotypic consequences of circZNF148 loss (Figure S18D–F), confirming that both ADRM1 and UCH37 are functionally required effectors downstream of circZNF148. Collectively, these findings establish that circZNF148 functions as a molecular scaffold that promotes the assembly of an HK1‐ADRM1 complex, thereby facilitating UCH37‐mediated deubiquitination of HK1. This tripartite assembly enables site‐specific removal of K11‐linked ubiquitin chains from HK1, shielding it from proteasomal degradation, ultimately enhancing HK1 stability and sustaining glycolytic metabolism and metastatic properties in TNBC cells.

### 3.7 circZNF148 Impairs CD8^+^ T Cell Function via Lactate‐Mediated Suppression

Having established that circZNF148 enhances glycolytic flux via HK1 stabilization, we next investigated whether this metabolic rewiring impacts the tumor immune microenvironment. A series of in vitro experiments were subsequently performed to investigate its impact on CD8^+^ T cell cytotoxicity. Flow cytometry‐based apoptosis assays were first performed to assess tumor cell death under different treatment conditions (Figure 6A). Co‐culture assays with MDA‐MB‐231 or MDA‐MB‐468 cells and human CD8^+^ T cells revealed that circZNF148 knockdown significantly enhanced tumor cell apoptosis, whereas circZNF148 overexpression conferred resistance to T cell‐mediated killing (Figure 6B, Figure S19A). These findings suggest that circZNF148 enables tumor cells to evade immune surveillance. Notably, patients with metastatic breast cancer, particularly those with lung metastases, often exhibit poor response to ICIs [26], suggesting the presence of immune resistance mechanisms in advanced disease. Given that circZNF148 is upregulated in metastatic lesions, we hypothesized that it contributes to therapy resistance via metabolic immunosuppression. Indeed, the highly metastatic 231/Lung subline exhibited significantly higher extracellular lactate levels compared to parental MDA‐MB‐231 cells (Figure S19B). Moreover, knockdown of circZNF148 in 231/Lung cells markedly reduced lactate accumulation in the supernatant (Figure S19B), linking circZNF148 expression to enhanced glycolytic output in aggressive cells.

**FIGURE 6:**
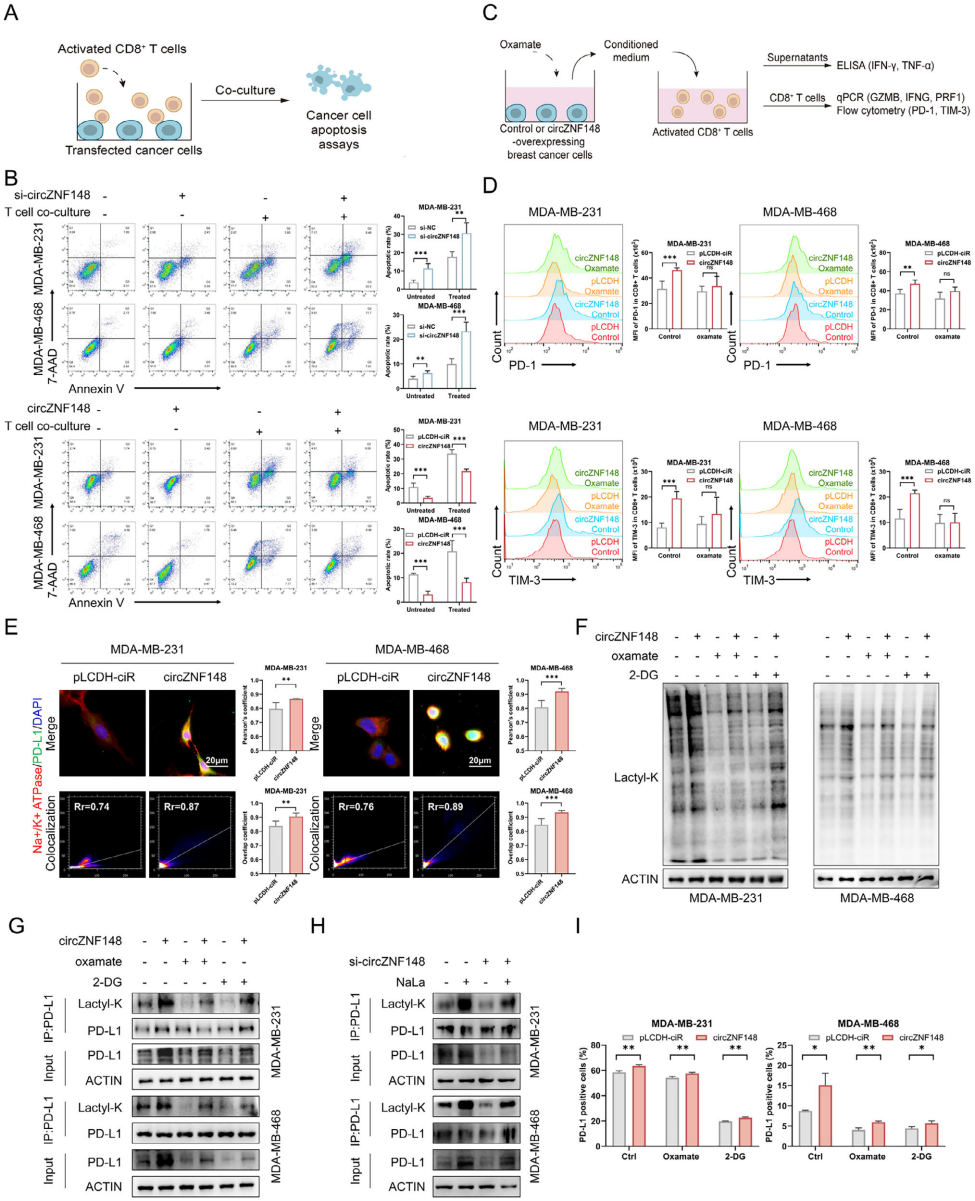
circZNF148 impairs CD8^+^ T cell cytotoxicity via lactate‐mediated PD‐L1 lactylation. (A) Schematic of the co‐culture apoptosis assay. (B) Flow cytometry quantification of apoptosis in TNBC cells co‐cultured with human CD8^+^ T cells under the indicated conditions. circZNF148 knockdown sensitizes, while its overexpression confers resistance to T cell‐mediated cytotoxicity. (C) Schematic of the experimental workflow for generating conditioned media (CM) and treating CD8^+^ T cells. (D) circZNF148‐induced lactate drives CD8^+^ T cell exhaustion. Flow cytometry analysis shows increased surface expression of the exhaustion markers PD‐1 and TIM‐3 on CD8^+^ T cells cultured in CM from circZNF148‐overexpressing cells. This effect is abrogated when lactate production is inhibited with oxamate. (E) Immunofluorescence staining confirming enhanced plasma membrane localization of PD‐L1 upon circZNF148 overexpression. Scale bars, 20 µm. (F) circZNF148 overexpression elevates global protein lactylation, an effect reversed by the glycolytic inhibitors oxamate or 2‐DG. (G) Immunoprecipitation of PD‐L1 followed by immunoblotting with anti‐lactyl‐K antibody shows enhanced PD‐L1 lactylation upon circZNF148 overexpression, which is attenuated by glycolytic inhibition. (H) Exogenous sodium lactate (NaLa, 20 mM) supplementation for 24 h rescues PD‐L1 lactylation levels in circZNF148‐knockdown cells. (I) Flow cytometry analysis shows the effects of glycolytic inhibitors and circZNF148 on surface PD‐L1. ns, no significance; *p < 0.05; **p < 0.01; ***p < 0.001.

Given that circZNF148 enhances glycolytic flux and lactate production, we wondered whether accumulated extracellular lactate contributes to CD8^+^ T cell dysfunction. To test this, conditioned media were collected from control or circZNF148‐overexpressing breast cancer cells treated with or without oxamate, and used to culture primary human CD8^+^ T cells (Figure 6C). Lactate assays confirmed that supernatants from circZNF148‐overexpressing cells contained significantly higher extracellular lactate levels compared to controls, and this increase was effectively suppressed by oxamate treatment (Figure S19C). Conversely, knockdown of circZNF148 reduced extracellular lactate production, and this reduction was partially rescued by ectopic overexpression of HK1 (Figure S19D), indicating that HK1 acts downstream of circZNF148 to drive glycolytic lactate production. ELISA analysis showed that CD8^+^ T cells exposed to high‐lactate supernatants from circZNF148‐overexpressing cells exhibited reduced secretion of key effector cytokines, including IFN‐γ and TNF‐α (Figure S19E). Consistently, qRT‐PCR revealed downregulation of *GZMB, IFNG, and PRF1* mRNA levels (Figure S19F), indicating impaired cytolytic capacity. Flow cytometry further demonstrated upregulated expression of immune checkpoint receptors PD‐1 and TIM‐3 on CD8^+^ T cells cultured in high‐lactate conditions (Figure 6D), consistent with an exhausted phenotype. Notably, treatment with oxamate decreased lactate accumulation in the supernatants and reversed these immunosuppressive effects: cytokine secretion was restored, cytotoxic gene expression recovered, and PD‐1/TIM‐3 upregulation was abrogated (Figure 6D, Figure S19E,F). To exclude the possibility that these outcomes were driven by pleiotropic or off‐target effects of the inhibitor on the immune cells, we evaluated CD8^+^ T cells treated directly with glycolysis inhibitors in the absence of tumor cells. CFSE staining and Annexin V/**7‐AAD** assays showed no significant impairment in proliferation or induction of apoptosis (Figure S19G,H). Furthermore, neither 2‑DG nor oxamate altered IFN‑γ and TNF‑α secretion (Figure S19I), *GZMB, IFNG, PRF1* mRNA levels (Figure S19J), or activation‑induced PD‑1/TIM‑3 expression (Figure S19K). These controls establish that the rescue of T cell function in our model is predominantly due to the depletion of tumor‐derived lactate, rather than direct pharmacological effects on the T cells. Additionally, conditioned media from circZNF148‐knockdown cells exerted the opposite effects: CD8^+^ T cells exhibited enhanced secretion of IFN‐γ and TNF‐α and reduced surface expression of PD‐1 and TIM‐3 (Figure S20A,B), reflecting improved functionality and reduced exhaustion. To verify whether lactate suppresses CD8^+^ T cells independently of low pH, we treated CD8^+^ T cells with sodium lactate (NaLa), HCl, or lactic acid (LA). None of these treatments caused a significant reduction in cell viability or increase in apoptosis (Figure S21A,B). Compared to the mild effects of the HCl‐acidified medium, treatment with NaLa significantly reduced cytokine secretion and cytotoxic gene expression, while upregulating exhaustion markers (Figure S21C–E). Lactic acid produced robust effects comparable to NaLa. This indicates that the lactate anion itself, rather than an acidic environment, is the primary driver of T cell dysfunction. Together, these results demonstrate that circZNF148 orchestrates an immunosuppressive microenvironment by elevating lactate levels, which directly suppresses CD8^+^ T cell function and promotes T cell exhaustion.

### 3.8 circZNF148 Stabilizes PD‐L1 Through Lysine Lactylation to Facilitate Immune Escape

Beyond metabolic suppression, we explored whether circZNF148 regulates intrinsic immune checkpoint pathways. Membrane‐localized PD‐L1 can directly bind PD‐1 on cytotoxic T cells to suppress antitumor immunity [27], and its expression in TNBC is associated with poor progression‐free and overall survival [28]. Notably, we found that the highly metastatic 231/Lung subline exhibited significantly elevated PD‐L1 protein levels and enhanced plasma membrane localization compared to parental MDA‐MB‐231 cells (Figure S22A–C), as confirmed by both flow cytometry and IF assays. Strikingly, circZNF148 overexpression in TNBC cells markedly increased both total and plasma membrane‐localized PD‐L1 (Figure 6E, Figure S22D,E), whereas circZNF148 knockdown reduced PD‐L1 expression at the total protein level and substantially diminished its surface presentation (Figure S22F–H). Recent studies have shown that lysine lactylation, a lactate‐dependent post‐translational modification, stabilizes PD‐L1 by blocking lysosomal degradation [29]. Given that circZNF148 enhances glycolysis and lactate production, we first assessed its impact on global protein lactylation. Pan‐lactyl‐lysine immunoblotting revealed elevated global lactylation levels in 231/Lung cells compared to parental lines, which was diminished upon circZNF148 knockdown (Figure S23A), indicating a link between circZNF148, hyperlactylated proteome and higher metastatic potential. CircZNF148 overexpression led to elevated global protein lactylation, and this effect was reversed upon pharmacological inhibition of glycolysis using oxamate or 2‐DG (Figure 6F). Consistently, exogenous lactate supplementation restored global lactylation level in circZNF148‐knockdown cells (Figure S23B,C), further linking circZNF148 to lactylation via metabolic reprogramming. We next focused on the effect of circZNF148 on PD‐L1 lactylation. Co‐IP assays demonstrated that circZNF148 overexpression enhances PD‐L1 lactylation at lysine residues in TNBC cells, whereas lactate inhibition using oxamate or 2‐DG attenuated this modification (Figure 6G, Figure S23D). Conversely, exogenous lactate supplementation restored PD‐L1 lactylation in circZNF148‐deficient cells (Figure 6H). Importantly, flow cytometry analysis revealed that the elevated membrane PD‐L1 induced by circZNF148 overexpression was significantly reduced upon treatment with glycolysis inhibitors (Figure 6I, Figure S23E). Conversely, the reduction in surface PD‐L1 caused by circZNF148 knockdown was rescued by exogenous NaLa supplementation (Figure S23F). These data further demonstrate that lactate‐driven lactylation is the essential mechanism stabilizing PD‐L1 on the cell membrane, thereby sustaining its immunosuppressive function. To exclude the confounding effect of extracellular acidification, we performed pH‐clamped experiments. Treatment with NaLa or lactic acid robustly induced global and PD‐L1‐specific lactylation, whereas the HCl‐acidified control failed to do so (Figure S23G,H), confirming that PD‐L1 lactylation is strictly driven by the lactate substrate, independent of pH changes. These data establish that circZNF148 couples metabolic activity to immune checkpoint regulation by promoting lactate‐driven PD‐L1 lactylation, stabilizing PD‐L1 and enhancing its membrane localization, thereby enhancing tumor cell immune evasion.

### 3.9 Targeting the circZNF148‐Glycolysis Axis Sensitizes Tumors to PD‐L1 Blockade In Vivo

We next evaluated whether circZNF148 modulates the response to PD‐L1 blockade in vivo using Hu‐PBL‐SCID TNBC xenografts established by intravenous infusion of human PBMCs followed by subcutaneous implantation of TNBC cells (Figure 7A). Mice bearing subcutaneous tumors derived from control or circZNF148‐overexpressing TNBC cells were treated with: isotype IgG control, anti‐PD‐L1 antibody durvalumab [12], or durvalumab combined with the glycolysis inhibitor 2‐DG. Flow cytometry analysis of tumor‐infiltrating immune cells and tumor cells revealed that circZNF148 overexpression led to elevated PD‐L1 on tumor cells and increased PD‐1 and TIM‐3 expression on CD8^+^ T cells (Figure 7B). While durvalumab monotherapy partially reversed these immunosuppressive features, the combination with 2‐DG achieved a more robust restoration of CD8^+^ T cell function (Figure 7B). Consistent with these immune changes, circZNF148 overexpression accelerated tumor growth, as reflected in increased tumor volume and weight (Figure 7C–E). Notably, combined treatment with durvalumab and 2‐DG produced the greatest inhibition of tumor growth. TUNEL assays further demonstrated reduced apoptosis in circZNF148‐overexpressing tumors, which was effectively restored by dual therapy (Figure 7F, Figure S24A). Histopathological examination (H&E) confirmed typical tumor morphology. ISH and IHC analyses revealed that circZNF148 overexpression correlated with increased expression of HK1, PD‐L1, and Ki67, consistent with its role in promoting glycolysis, proliferation, and immune evasion (Figure 7G, Figure S24B). Notably, combination treatment with durvalumab and 2‑DG suppressed these markers more effectively than durvalumab alone, supporting an enhanced effect of metabolic inhibition and immune checkpoint blockade. To evaluate the in vivo relevance between circZNF148 expression and breast cancer metastasis, we established experimental lung metastasis models via tail vein injection of control or circZNF148‐overexpressing TNBC cells in immunodeficient mice. Strikingly, circZNF148 overexpression dramatically elevated the number and size of lung metastatic nodules (Figure S24C), underscoring its role in promoting metastatic potential. Collectively, these results demonstrate that circZNF148 drives tumor progression by coupling enhanced glycolysis to PD‐L1‐mediated immune evasion, highlighting the therapeutic potential of combining glycolytic inhibitors with ICIs in circZNF148‐high TNBC.

**FIGURE 7:**
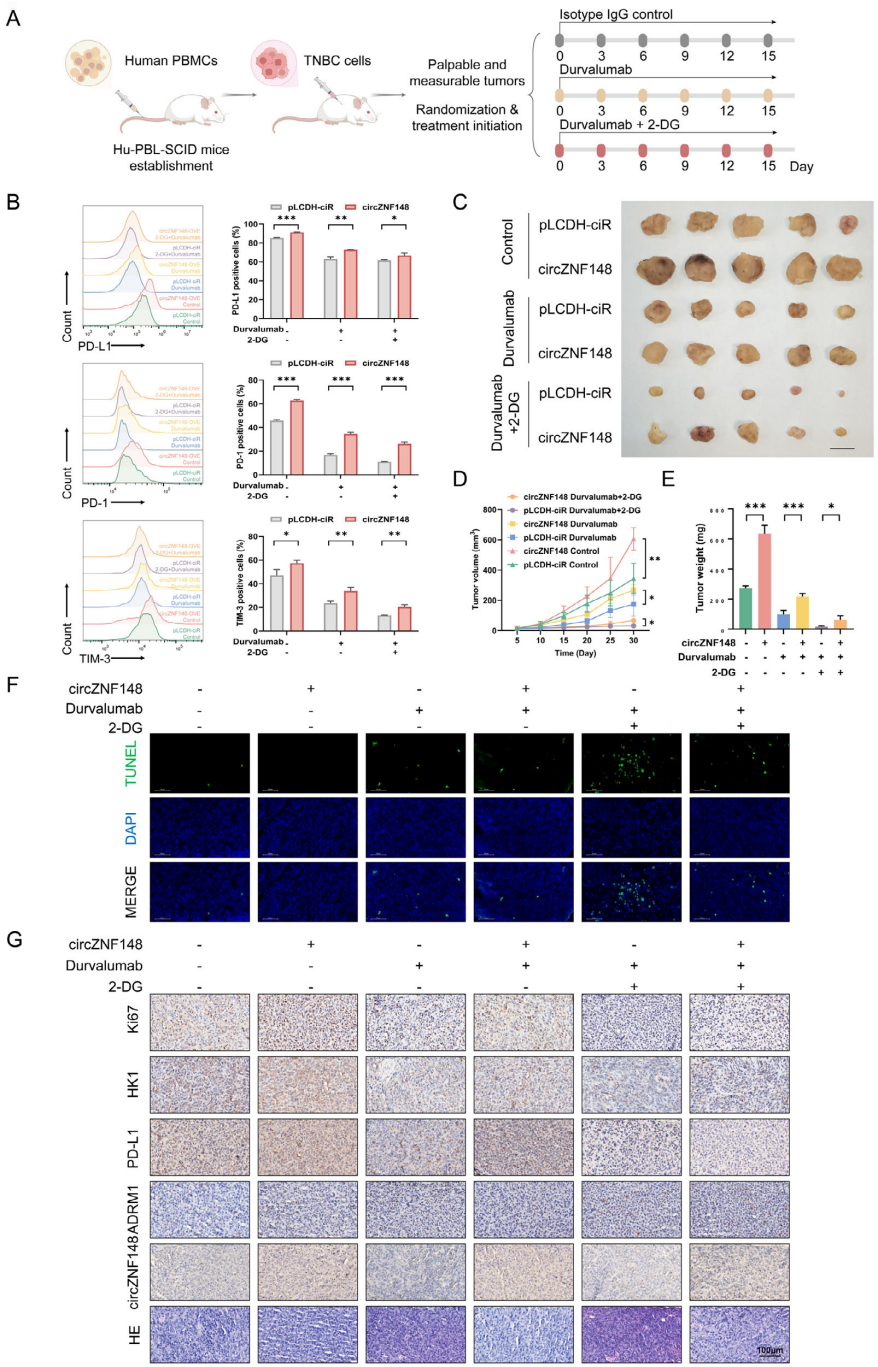
circZNF148 promotes immune escape and resistance to PD‐L1 blockade in vivo. (A) Schematic of the humanized xenograft model. (B) Flow cytometry analysis of tumor‐infiltrating immune cells and tumor cells in the indicated groups. (C‐E) circZNF148 overexpression promotes tumor growth: (C) Representative tumor images. Scale bar, 1 cm. (D) Tumor growth curves. (E) Final tumor weights. (F) TUNEL staining for apoptosis in tumor xenografts. Scale bars, 50 µm. (G) Representative images of in situ hybridization (ISH) for circZNF148 and immunohistochemistry (IHC) for HK1, ADRM1, PD‐L1, and Ki67 in tumor xenografts. Scale bars, 100 µm. *p < 0.05; **p < 0.01; ***p < 0.001.

### 3.10 The Clinical Relevance of circZNF148 in Breast Cancer

To evaluate the clinical relevance of circZNF148 in breast cancer, we first assessed its expression in paired primary tumor and adjacent normal breast tissues using qRT‐PCR. circZNF148 was significantly upregulated in tumor tissues compared to matched normal tissues (Figure 8A), indicating its tumor‐associated expression pattern. This finding was further validated by ISH on formalin‐fixed paraffin‐embedded (FFPE) tissues from TNBC patients. ISH signals for circZNF148 were predominantly localized in the cytoplasm and stronger in tumor regions compared to adjacent histologically normal epithelium (Figure 8B).

**FIGURE 8:**
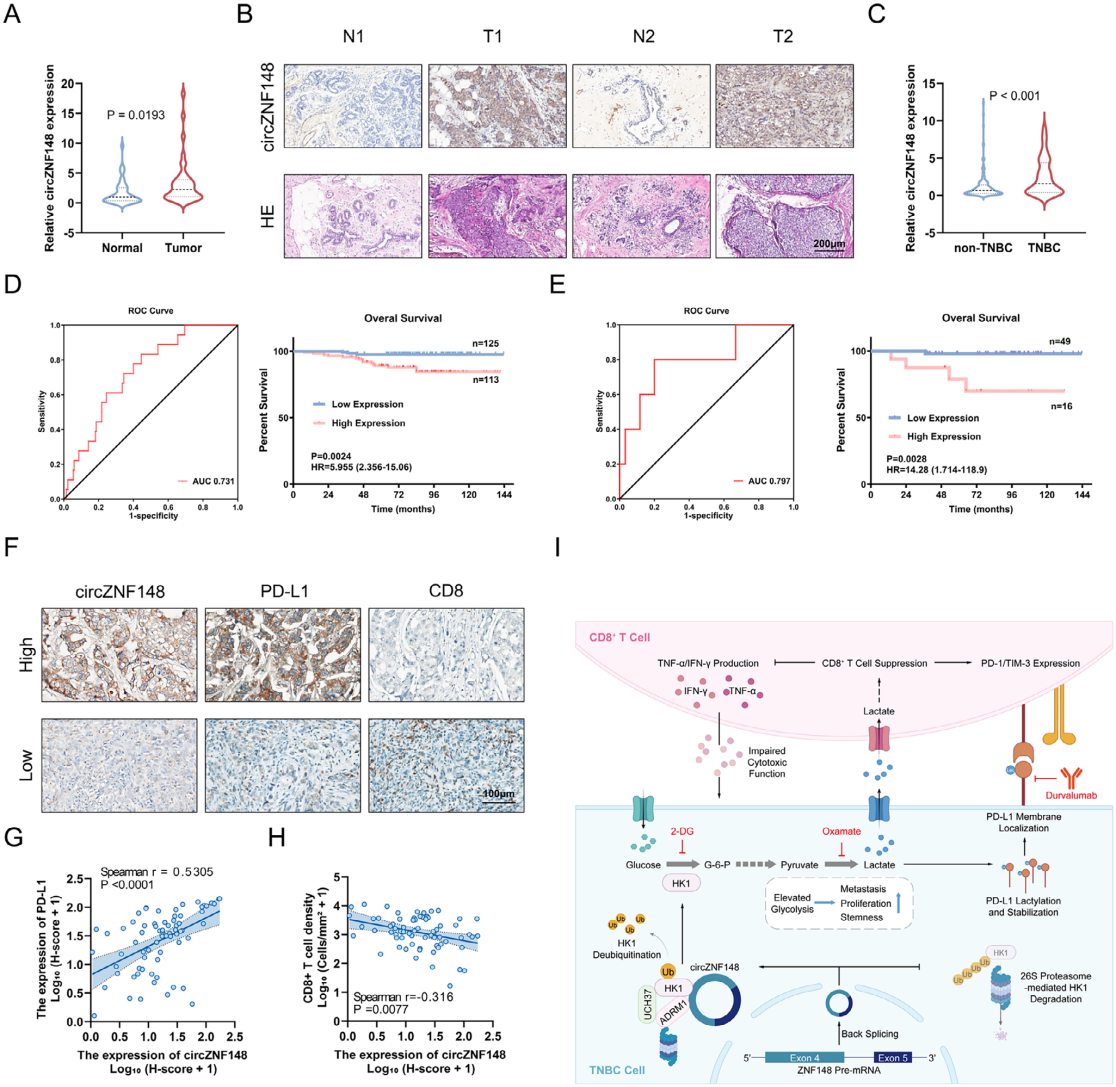
Clinical significance of circZNF148 in breast cancer. (A) qRT‐PCR analysis of circZNF148 expression in paired primary breast tumor (T) and adjacent normal (N) tissues. (B) Representative images of in situ hybridization (ISH) for circZNF148 and HE staining in FFPE sections from TNBC patients. Scale bars, 200 µm. (C) The expression of circZNF148 in TNBC and non‐TNBC tissues. (D) Receiver operating characteristic (ROC) curve analysis in a cohort of 238 breast cancer patients and the Kaplan‐Meier curve comparing overall survival of patients with high vs. low circZNF148 expression in the full cohort. *p*‐values were calculated by the log‐rank test. (E) Receiver operating characteristic (ROC) curve analysis in the TNBC subgroup and the Kaplan‐Meier curve comparing overall survival of patients with high vs. low circZNF148 expression in the cohort. *p*‐values were calculated by the log‐rank test. (F) Representative ISH and IHC images showing the expression of circZNF148, PD‐L1, and CD8 in TNBC tissue sections. Scale bars, 100 µm. (G) Correlation analysis between circZNF148 ISH scores and PD‐L1 IHC scores in TNBC patients. (H) Correlation analysis between circZNF148 ISH scores and CD8^+^ T cell density in TNBC patients. (I) Schematic illustrating how circZNF148 upregulation leads to HK1 stabilization, enhancing glycolysis and driving metastatic dissemination and immune suppression in TNBC.

We next correlated circZNF148 expression with clinicopathological features in a cohort of 238 breast cancer patients. To establish a comparative baseline and validate subtype specificity, we first compared the expression of circZNF148 between TNBC (n = 65) and non‐TNBC (n = 173) patients within the cohort. As shown in Figure 8C, circZNF148 expression was significantly higher in TNBC tissues than in non‐TNBC tissues. To define a clinically relevant threshold for stratification, ROC curve analysis was performed, yielding an AUC of 0.731 (95% CI: 0.626–0.820). The optimal cut‐off value was determined by the maximum Youden index, and patients were subsequently classified into high or low circZNF148 expression groups according to this validated threshold. High circZNF148 expression was closely associated with distant metastasis status (P < 0.05; Table S4). Kaplan‐Meier survival analysis revealed that patients with high circZNF148 expression had significantly shorter overall survival (OS) compared to those with low expression (Figure 8D; log‐rank P = 0.0024; HR = 5.96, 95% CI: 2.356–15.06). Given the significantly elevated baseline expression of circZNF148 in TNBC tissues, we further re‐derived a TNBC‐specific optimal cut‐off exclusively within the TNBC subgroup using the same Youden index method (Figure 8E; AUC = 0.797, 95% CI: 0.557–0.973). The Kaplan‐Meier analysis restricted to the TNBC subgroup revealed a markedly higher HR (Figure 8E; HR = 14.28, 95% CI: 1.714–118.9, P = 0.0028), indicating that circZNF148 may be a particularly potent driver of progression in TNBC. Significantly, univariate and multivariate Cox regression analyses confirmed that high circZNF148 expression is an independent predictor of poor prognosis (HR = 1.226, 95% CI: 1.030–1.459, P = 0.022; Table S5). In summary, circZNF148 serves as an independent prognostic biomarker in breast cancer, with high expression significantly associated with tumor progression and poor survival outcomes.

To further validate the clinical significance of circZNF148 in modulating the immune microenvironment, we analyzed its correlation with PD‐L1 expression and CD8^+^ T cell infiltration in TNBC tissues (Figure 8F). Quantitative analysis revealed a positive correlation between circZNF148 and PD‐L1 protein levels (Figure 8G). Conversely, high circZNF148 expression was inversely associated with the density of tumor‐infiltrating CD8^+^ T cells (Figure 8H). These findings suggest that circZNF148 contributes to an immunosuppressive phenotype in TNBC patients, consistent with our experimental models of immune evasion.

## 4 Discussion

TNBC is the most aggressive and therapeutically challenging subtype of breast cancer, characterized by pronounced heterogeneity, a high propensity for metastasis, limited treatment options, and poor clinical outcomes [30, 31]. At the biological level, TNBC is characterized by extensive metabolic reprogramming and a strong reliance on glycolysis to support rapid proliferation, survival under nutrient stress, and metastatic dissemination [32]. While the role of metabolic dysregulation in tumor progression is well established, the upstream molecular regulators that orchestrate this reprogramming in TNBC remain incompletely understood. CircRNAs have emerged as pivotal players in post‐transcriptional and post‐translational regulation of cancer metabolism [33], yet their functional roles in shaping the metabolic landscape of TNBC remain largely unexplored. In this study, we identify a novel circRNA, circZNF148, as a critical driver of metabolic reprogramming in TNBC. We demonstrate that circZNF148 significantly enhances glycolytic flux and lactate production, enabling TNBC cells to thrive in glucose‐deprived microenvironments, a common feature of rapidly growing tumors, which aligns with prior studies implicating circular RNAs in metabolic regulation [34]. This metabolic rewiring induced by circZNF148 enhances multiple malignant phenotypes of TNBC, including migration, invasion, cancer stem‐like properties, and immune evasion (Figure 8I). Notably, elevated circZNF148 expression in TNBC tissues correlates with aggressive phenotypes and unfavorable clinical outcomes, highlighting its potential as a prognostic biomarker and a therapeutic target.

Given the prominent role of circZNF148 in metabolic reprogramming, we next explored how circZNF148 itself is upregulated in TNBC, particularly under glucose‐deprived conditions. Using bioinformatic prediction and expression screening, we identified IGF2BP2 as a critical upstream regulator of circZNF148 biogenesis. IGF2BP2 is a well‐known RNA‐binding protein that often functions as a stabilizer or m^6^A reader to modulate cancer progression [35, 36]. We demonstrate that IGF2BP2 binds to the intronic flanking regions of circZNF148 and promotes its accumulation. This regulation occurs at the level of cyclization rather than RNA stability, as evidenced by actinomycin D assays. Moreover, our data show that low‐glucose stress induces IGF2BP2 expression, and that IGF2BP2 knockdown abrogates the low‐glucose‐induced increase in circZNF148. This suggests that metabolic stress, a hallmark of the tumor microenvironment, can actively reprogram circRNA expression by inducing specific RBPs. Low glucose is known to activate various stress signaling pathways, such as AMPK and mTOR [37, 38]. Whether these pathways directly control IGF2BP2 transcription or post‐transcriptional regulation requires further study. Nevertheless, our findings provide a mechanistic link between metabolic stress and circRNA biogenesis, highlighting that circRNAs like circZNF148 may serve as sensors or effectors of nutrient availability in aggressive TNBC cells.

A central mechanistic insight of our study is the identification of circZNF148 as a critical stabilizer of HK1, the first rate‐limiting enzyme in glycolysis that catalyzes the conversion of glucose to glucose‐6‐phosphate, a key commitment step in glucose metabolism [39]. Although HK1 turnover is known to be regulated by ubiquitin‐mediated proteasomal degradation, a process counteracted by deubiquitinating enzymes (DUBs) and their adaptors such as ADRM1 [40, 41], the precise regulatory mechanisms governing HK1 stability in TNBC remain poorly understood. Through mass spectrometry and RNA pull‐down assays, we identified both HK1 and ADRM1 as binding partners of circZNF148. ADRM1 is an ubiquitin receptor subunit of the 19S proteasomal regulatory particle that recognizes polyubiquitinated substrates and facilitates their delivery to the proteasome [42]. It also serves as a platform for recruiting UCH37, a proteasome‐associated DUB of the ubiquitin C‐terminal hydrolase family, which removes ubiquitin chains from substrate proteins [43]. Notably, circZNF148 physically associates with ADRM1, whereas no association between circZNF148 and UCH37 was detected in our RNA pull‐down assays. Instead, co‐IP and immunofluorescence analyses revealed that circZNF148 promotes the association between HK1 and ADRM1 and enables the subsequent recruitment of UCH37 to HK1 in a circZNF148‐dependent manner. Mechanistically, ADRM1 recruits and activates UCH37 to cleave degradative ubiquitin chains [24, 42, 44, 45]. In the presence of circZNF148, this ADRM1‐UCH37 complex removes K11‐linked polyubiquitin chains from HK1, a modification increasingly recognized as a potent signal for proteasomal degradation [46, 47, 48, 49, 50]. This aligns with growing evidence that DUBs such as USP19, USP10, and USP1 stabilize oncogenic or metabolic regulators by counteracting K11‐linked ubiquitination [46, 47, 49]. By shielding HK1 from this degradation signal, circZNF148 significantly extends its protein half‐life, thereby reinforcing glycolytic flux and promoting lactate accumulation. Importantly, circZNF148 did not affect the mRNA or protein levels of ADRM1 or UCH37, indicating that its role is not in regulating their expression but rather in orchestrating their functional engagement with HK1. Functional validation further supports the essential role of ADRM1 and UCH37 in mediating the effect of circZNF148. Overexpression of either ADRM1 or UCH37 reduced K11‐linked ubiquitination of HK1, prolonged its protein half‐life, and rescued HK1 expression in circZNF148‐deficient cells, thereby restoring glycolytic activity, stemness, and metastatic capacity. Conversely, knockdown of ADRM1 or UCH37 abolished the stabilizing effect on HK1 and oncogenic phenotypes induced by circZNF148 overexpression. Our domain mapping and in silico 3D modeling provide a precise structural basis for this coordinated assembly. HK1, circZNF148, and UCH37 occupy distinct, non‑overlapping domains on ADRM1 (N‑terminal, middle, and C‑terminal, respectively), enabling simultaneous assembly without steric hindrance and positioning UCH37 for recruitment to the HK1‑ADRM1 complex. These findings define a novel circZNF148‐ADRM1‐UCH37 regulatory model that stabilizes HK1 through selective deubiquitination, wherein circZNF148 functions as a molecular scaffold bridging HK1 and ADRM1 to facilitate UCH37 recruitment and subsequent removal of K11‐linked ubiquitin chains. This post‐translational regulatory mechanism further deepens our understanding of how metabolic enzymes are stabilized in cancer, and underscores the emerging role of circRNAs as functional scaffolds in protein complex assembly.

The stabilization of HK1 by circZNF148 leads to sustained glycolytic activation and robust lactate accumulation. Beyond serving as a metabolic end product, lactate functions as a key immunometabolite and signaling molecule that profoundly shapes the tumor microenvironment [51]. It acidifies the extracellular milieu, destabilizes the extracellular matrix, promotes angiogenesis, and suppresses immune surveillance [39]. Our data show that elevated lactate levels directly impair CD8^+^ T cell function by suppressing the secretion of critical effector cytokines (TNF‐α and IFN‐γ), downregulating cytotoxic effector molecules (*PRF1, GZMB, IFNG*), and upregulating the expression of exhaustion markers (PD‐1, TIM‐3). Importantly, pharmacological inhibition of lactate production using oxamate restored T cell activity, confirming lactate's role as a pivotal immunometabolic checkpoint. These observations align with prior studies in melanoma and lung cancer, showing that lactate accumulation contributes to immune suppression and resistance to PD‐1 blockade [52, 53].

Another striking finding of our study is the direct link between circZNF148‐driven glycolysis and immune checkpoint regulation through protein lysine lactylation, a recently discovered post‐translational modification (PTM). Previous reports have established that enhanced glycolytic activity can upregulate PD‐L1 in tumor cells [54, 55], while glycolysis inhibitors can suppress PD‐L1 levels or disrupt its glycosylation to reverse immunosuppression [56, 57]. We extend these concepts to TNBC by providing upstream mechanistic insight. CircZNF148‐mediated lactate accumulation led to elevated PD‐L1 lactylation, which enhanced its plasma membrane localization and capacity to suppress CD8^+^ T cell cytotoxicity, thereby creating an immunosuppressive niche. Silencing circZNF148 reduced lactate burden, diminished PD‐L1 lactylation and expression, and sensitized tumors to durvalumab therapy, underscoring its role as a master regulator at the metabolic‐immune interface. This discovery provides a mechanistic explanation for the observed immunosuppressive microenvironment in glycolytically active tumors and establishes lactate‐mediated lactylation as a critical bridge between cancer metabolism and immune suppression.

Despite these robust findings, the precise lysine residue(s) of PD‐L1 targeted by lactylation downstream of the circZNF148‐lactate axis in our TNBC model remain to be identified. Recent studies have uncovered the complexity of PD‐L1 lactylation, demonstrating that this modification occurs at distinct lysine residues depending on the cellular context and drives diverse functional outcomes. For instance, lactylation at K280 in non‐small cell lung cancer stabilizes PD‐L1 by evading HUWE1‐mediated proteasomal degradation [58], while lactylation at K271 in colorectal cancer delays its lysosomal degradation [29]. Conversely, in liver cancer, PD‐L1 lactylation at K189 suppresses tumor growth, whereas its delactylation serves as a dynamic switch for subcellular localization, promoting nuclear translocation and tumor progression [59]. Given that lactylation sites and their corresponding mechanisms are highly context‐dependent, it is possible that TNBC exhibits unique modification sites. Future studies employing liquid chromatography‐tandem mass spectrometry (LC‐MS/MS) to map the precise lactylation sites on PD‐L1 in TNBC, combined with site‐directed mutagenesis to validate their functional relevance, will be essential to fully elucidate how lactylation stabilizes PD‐L1 at the cell surface.

From a therapeutic standpoint, our findings are expected to address a critical unmet need in TNBC management. Despite advances in ICIs, monotherapy yields durable responses in only ∼20% of TNBC patients and less than 10% in metastatic settings [60], largely due to the profoundly immunosuppressive TME [61]. The glycolysis‐derived lactate not only inhibits the proliferation and cytotoxic activity of effector T cells [62] but also supports pre‐metastatic niche formation, enabling disseminated tumor cells to evade immune surveillance and colonize distant organs such as the lungs [63, 64]. The broader clinical relevance of circRNA‐mediated immune regulation in breast cancer is increasingly recognized. For instance, circHIPK3 is associated with poor response to immunotherapy [65], hsa_circ_0067842 stabilizes PD‐L1 via the HuR/CMTM6 axis [66], and circFGFR4 reduces CD8^+^ T cell infiltration and confers resistance to ICIs in TNBC [67]. Moreover, experimental depletion of oncogenic circRNAs such as circDnmt1 further supports their therapeutic potential, although challenges related to specificity, delivery, and toxicity remain to be addressed [68]. Our findings also demonstrated the close relevance between circZNF148 expression and response to durvalumab in TNBC. These findings, together with our work, position circRNAs as central nodes in the cancer immunity cycle. Given their inherent stability and detectability in biofluids [65], circRNAs are promising candidates for non‐invasive biomarkers. In this context, circZNF148 emerges not only as a functional driver but also as a liquid biopsy candidate for monitoring disease progression and immunotherapy efficacy in TNBC.

Therapeutically, our findings support the growing rationale for combining metabolic inhibitors with ICIs. Preclinical studies have shown that agents targeting key glycolytic enzymes, such as 2‐DG, a glucose analog competitively inhibiting hexokinase [69], and oxamate, a pyruvate analog inhibiting lactate dehydrogenase [70], exhibit significant immunomodulatory effects, including restoration of T cell function and enhancement of anti‐tumor immunity, highlighting a promising therapeutic strategy for aggressive TNBC. Notably, we show that overexpression of circZNF148 drives resistance to durvalumab, consistent with its role in establishing an immunosuppressive tumor microenvironment through HK1 stabilization, lactate accumulation, and PD‐L1 lactylation. While the addition of 2‐DG sensitizes tumors to immunotherapy and enhances treatment efficacy, this combination is less effective in circZNF148‐overexpressing tumors, which still exhibit residual metabolic activity and partial immune evasion, likely due to persistent HK1 stability and incomplete suppression of lactate production. This residual resistance underscores the limitations of broad glycolytic inhibition and highlights the compensatory mechanisms that may bypass enzyme‐targeted drugs.

In contrast, targeting circZNF148 might be a more precise and tumor‐selective approach by disrupting the upstream oncogenic driver of glycolytic reprogramming, potentially minimizing off‐target effects while maximizing therapeutic synergy with immunotherapy. Strategies such as antisense oligonucleotides (ASOs), siRNAs, or CRISPR‐based approaches may enable selective targeting of circZNF148, combined with PD‐1/PD‐L1 blockade, offering a feasible dual‐pronged approach to simultaneously disrupt tumor‐intrinsic metabolism and tumor‐extrinsic immunosuppression. Despite this immense promise, the clinical translation of these RNA‐targeted modalities faces several formidable hurdles [71, 72]. First, achieving adequate delivery efficiency into dense solid tumors remains a significant barrier, compounded by the challenge of facilitating intracellular endosomal escape. Second, ensuring robust tumor specificity is critical to minimize systemic toxicity and protect healthy tissues. Third, the potential for off‐target effects, such as unintended widespread gene silencing by siRNAs/ASOs or off‐target genomic cleavage by CRISPR systems, requires rigorous sequence optimization. To overcome these translational bottlenecks, future investigations should prioritize the development of advanced, precision‐guided delivery platforms. For instance, engineering tumor‐targeted lipid nanoparticles (LNPs) or utilizing antibody‐oligonucleotide conjugates (AOCs) directed against TNBC‐specific surface markers might enhance the bioavailability and in vivo efficacy while improving safety [73, 74]. Future studies should further explore whether circZNF148 modulates HK1 in other glycolysis‐dependent cancers and validate its utility as a predictive biomarker for response to PD‐1/PD‐L1 blockade in TNBC patients.

In this study, our analysis of TNBC clinical specimens demonstrated that circZNF148 is positively associated with PD‐L1 expression and negatively correlated with CD8^+^ T cell density. While our clinical cohort and in vivo humanized models support the immunomodulatory role of circZNF148, we acknowledge certain limitations regarding our experimental models. First, although the Hu‐PBL‐SCID mouse model facilitated the evaluation of CD8^+^ T cell‐mediated responses to PD‐L1 blockade, it primarily reconstitutes human lymphocytes and lacks a fully functional human myeloid compartment, such as tumor‐associated macrophages (TAMs) and myeloid‐derived suppressor cells (MDSCs). Given that lactate accumulation can also influence myeloid cell polarization [75], our current model may not capture the complete spectrum of the immunosuppressive network orchestrated by the circZNF148‐glycolysis axis. Future studies employing more advanced humanized immune system models [76], such as CD34^+^ hematopoietic stem cell‐engrafted mice or immune‐competent syngeneic models, will be necessary to further elucidate its impact on the broader tumor immune contexture. Second, direct clinical data correlating circZNF148 expression with the objective response rate (ORR) or survival outcomes in TNBC patients actively receiving ICIs are currently lacking. Due to the limited availability of clinical cohorts with matched circRNA profiling and ICI follow‐up, future prospective studies are warranted to definitively establish circZNF148 as a predictive biomarker for immunotherapy response in the clinic. Additionally, although our analysis revealed the association between elevated circZNF148 expression and poor prognosis of breast cancer patients, its prognostic value in the TNBC subgroup requires further validation in larger, independent cohorts.

## 5 Conclusion

In this study, we identified and functionally characterized circZNF148 as a critical regulator orchestrating metabolic reprogramming and immune evasion in TNBC. CircZNF148 is elevated in TNBC tissues and correlates with poor prognosis. Mechanistically, circZNF148 stabilizes HK1 by recruiting the ubiquitin receptor ADRM1 to suppress K11‐linked ubiquitination, thereby prolonging HK1 protein half‐life. This post‐translational regulation drives sustained glycolytic activation and lactate accumulation, which promotes PD‐L1 lysine lactylation. Lactylated PD‐L1 exhibits enhanced stability, membrane localization, and immune checkpoint function, ultimately suppressing CD8^+^ T cell activity. Notably, combining glycolytic inhibition with durvalumab produced greater tumor growth inhibition than either treatment alone. Our findings uncover a key role for circZNF148 in coupling metabolic and immune dysregulation in TNBC, providing a strong rationale for combined therapeutic strategies that co‐target tumor metabolism and immune checkpoints to improve patient outcomes.

## CRediT Authorship Contribution Statement

Conception and design: Y. H. Jin, W. J. Zhao, Y. R. Liang, Q. F. Yang; Acquisition of data: Y. H. Jin, W. J. Zhao, L. Wang, J. N. Wang, K. Y. Zhao, D. Luo, Y. F. Wang, Y. X. Yang, J. Z. Yang; Analysis and interpretation of data: Y. H. Jin, W. J. Zhao, L. Wang, J. N. Wang, K. Y. Zhao, D. Luo, Y. F. Wang, Y. X. Yang, J. Z. Yang, Y. M. Li, T. Chen, D. W. Han, Z. K. Wang; Sample collection: X. Y. Li, B. Chen, L. J. Wang; Study supervision: X. Y. Li, B. Chen, L. J. Wang, Y. R. Liang, Q. F. Yang; Writing, review, and/or revision of the manuscript: Y. H. Jin, W. J. Zhao, Y. R. Liang, Q. F. Yang. All authors read and approved the final manuscript.

## Funding

This work was supported by Special Foundation for Taishan Scholars (No. tstp20250509, tsqn202507348), National Natural Science Foundation of China (No. 82573211, 82373005, 82573252), Shandong Provincial Natural Science Foundation (No. ZR2024MH242), and Young Talent of Lifting engineering for Science and Technology in Shandong, China (No. SDAST2025QTA089).

## Conflicts of Interest

The authors declare no conflicts interest.

## Supporting information




**Supporting File 1**: advs77030‐sup‐0001‐SuppMat.docx.

**Supporting File 2**: advs77030‐sup‐0002‐SuppTables.docx.

## Data Availability

The data that support the findings of this study are available from the corresponding author upon reasonable request.
