# Supplementary material for: circZNF148 Drives Glucose Metabolism Reprogramming to Enhance Metastasis and Immune Evasion via HK1 Stabilization in Triple‐Negative Breast Cancer
Source: Adv Sci (Weinh). 2026 Aug 7;13(60):e77030. doi: 10.1002/advs.77030 (PMC13449536; doi:10.1002/advs.77030)
Supplement: Supplementary file 1 [file ADVS-13-e77030-s001.docx]

**Supplementary Figures**

**Figure S1. Selection and initial characterization of circZNF148 in metastatic and glycolytic models.** (A) Analysis of glycolytic metabolites in parental MDA-MB-231 cells versus the lung-tropic metastatic subline (231/Lung). (B-C) Once MDA-MB-231 and MDA-MB-468 cells had attached, the medium was replaced with either low-glucose (1 g/L) or normal-glucose (4.5 g/L) DMEM. The pH was determined with a pH meter, while cell viability was assessed via MTT assay immediately upon replacement (0 h) and after a 24-h incubation. (D) **qRT-PCR measuring circ7895 and circ7943 expression in low-glucose-treated (24 h) and lung metastasis-prone cell models.** (E) Fluorescence in situ hybridization (FISH) analysis of circZNF148 expression in MDA-MB-231 and MDA-MB-468 cells under normal glucose or low glucose (24 h) conditions. Scale bars, 20 μm. (F) circZNF148 expression in breast cancer cell lines compared to the normal mammary epithelial cell line. ns, no significance; *P < 0.05; **P < 0.01; ***P < 0.001.

**Figure S2. circZNF148 knockdown specifically reduces circular RNA expression and inhibits TNBC cell proliferation and survival.** (A) **Schematic representation of circZNF148 back-splicing and the design of siRNAs specifically targeting the back-splice junction.** (B) Efficient and specific knockdown of circZNF148 using siRNAs targeting the back-splice junction, without affecting the mRNA expression of its linear host gene *ZNF148*, in MDA-MB-231, MDA-MB-468 and 231/Lung cells. (C) MTT assays measuring cell proliferation upon circZNF148 knockdown. (D) EdU incorporation assays measuring DNA replication upon circZNF148 knockdown. Scale bars, 200 μm. (E) Colony formation assays measuring clonogenic survival upon circZNF148 silencing. (F) Flow cytometry analysis of apoptosis following circZNF148 knockdown. ns, no significance; *P < 0.05; **P < 0.01; ***P < 0.001.

**Figure S3. circZNF148 knockdown attenuates the migratory and invasive capacities of TNBC cells.** (A) Wound healing assays measuring migratory abilities of MDA-MB-231, MDA-MB-468 and 231/Lung cells following circZNF148 knockdown. Quantification of the relative wound closure area is presented below. Scale bars, 200 μm. (B) Transwell assays measuring migratory and invasive abilities of MDA-MB-231, MDA-MB-468, and 231/Lung cells following circZNF148 knockdown. Scale bars, 200 μm. (C) Efficient and specific knockdown of circZNF148 using siRNAs targeting the back-splice junction, without affecting the mRNA expression of its linear host gene *ZNF148*, in BT-549 cells. (D) Transwell assays measuring migratory and invasive abilities of BT-549 cells following circZNF148 knockdown. Scale bars, 200 μm. ns, no significance; *P < 0.05; **P < 0.01; ***P < 0.001.

**Figure S4. Validation of circZNF148 overexpression and its impact on proliferation and apoptosis.** (A) qRT-PCR confirmation of ectopic circZNF148 overexpression without altering linear *ZNF148* mRNA levels. (B) MTT assays measuring cell proliferation ability upon circZNF148 overexpression. (C) EdU incorporation assays measuring DNA replication upon circZNF148 overexpression. Scale bars, 200 μm. (D) Flow cytometry analysis of apoptosis using Annexin V/**7-AAD** staining measuring apoptotic rates following circZNF148 overexpression. ns, no significance; *P < 0.05; **P < 0.01; ***P < 0.001.

**Figure S5. circZNF148 overexpression enhances the migratory and invasive phenotypes of TNBC cells.** (A) Wound healing assays measuring migratory abilities upon circZNF148 overexpression in MDA-MB-231 and MDA-MB-468 cells. Scale bars, 200 μm. (B) Transwell assays measuring migratory and invasive abilities upon circZNF148 overexpression in MDA-MB-231 and MDA-MB-468 cells. Scale bars, 200 μm. (C) qRT-PCR confirmation of circZNF148 overexpression without altering linear *ZNF148* mRNA levels. ns, no significance; *P < 0.05; **P < 0.01; ***P < 0.001.

**Figure S6. circZNF148 is essential for maintaining cancer stem cell properties.** (A) Intracellular lactate, ATP, and G6P levels were measured upon circZNF148 knockdown in BT-549 cells. (B) Sphere formation assays measuring sphere formation capacity upon circZNF148 knockdown. Scale bars, 50 μm. (C) Extreme limiting dilution analysis was performed upon circZNF148 knockdown and stem cell frequency was calculated via ELDA. (D) Flow cytometry analysis measuring ALDH⁺ proportions or CD44⁺CD24⁻ cancer stem-like subpopulations in breast cancer cells upon circZNF148 knockdown. *P < 0.05; **P < 0.01; ***P < 0.001.

**Figure S7. circZNF148 overexpression enhances glycolytic activity and stemness.** (A-D) Extracellular acidification rate (ECAR), intracellular ATP, G6P, and lactate were measured upon circZNF148 overexpression. (E) Sphere formation assays measuring sphere formation capacity upon circZNF148 overexpression. Scale bars, 50 μm. (F) Extreme limiting dilution analysis was performed upon circZNF148 overexpression and stem cell frequency was calculated via ELDA. (G) Flow cytometry analysis measuring ALDH⁺ proportions in MDA-MB-231 cells and CD44⁺CD24⁻ cancer stem-like subpopulations in MDA-MB-468 cells upon circZNF148 overexpression. **P < 0.01; ***P < 0.001.

**Figure S8. circZNF148 regulates the expression of key metabolic, EMT, and stemness markers.** (A) Western blot analysis showing the effects of circZNF148 knockdown and overexpression on the protein levels of glycolytic enzymes. (B) Western blot analysis showing the effects of circZNF148 knockdown and overexpression on the protein levels of EMT factors. (C) Western blot analysis showing the effects of circZNF148 knockdown and overexpression on the protein levels of stemness markers.

**Figure S9. circZNF148 mediates migration, invasion, stemness, and organoid response to glycolysis inhibition in TNBC.** (A) Following MDA-MB-231 and MDA-MB-468 cell attachment, the pH of the culture medium was measured immediately after medium change (0 h) and after 24 h of treatment with vehicle control, 2-DG (20 mM), or oxamate (10 mM). (B) Quantification of transwell migration (left) and invasion (right) assays. Transfected cells were treated with vehicle control or 2‑DG. (C) Quantification of transwell migration (left) and invasion (right) assays. Transfected cells were treated with vehicle control or oxamate. (D) Quantification of spheroid assays. Transfected cells were treated with vehicle control, 2-DG, or oxamate. (E) Quantification of patient‑derived organoid (PDO) models upon vehicle control, 2-DG, or oxamate treatment. ns, no significance; *P < 0.05; **P < 0.01; ***P < 0.001.

**Figure S10. IGF2BP2-mediated biogenesis of circZNF148.** (A) Bioinformatics analysis of RNA-binding proteins (RBPs) binding to circZNF148. (B) qRT-PCR analysis of transfection efficiency following knockdown or overexpression of IGF2BP2, KHDRBS3, and YBX1. (C) qRT-PCR analysis of circZNF148 expression following knockdown or overexpression of IGF2BP2, KHDRBS3, and YBX1. (D) RIP-qPCR assay confirming the direct binding of IGF2BP2 to the flanking intron sequences of circZNF148. (E-F) Cells were transfected with si-NC or si-IGF2BP2, and cultured in normal glucose medium or low glucose (1 g/L, 24 h). The mRNA (E) and protein (F) levels of IGF2BP2 were detected under the indicated conditions. (G) The expression of circZNF148 was evaluated under the indicated conditions. (H) MDA-MB-231 and MDA-MB-468 cells were transfected with si‑NC or si‑IGF2BP2, and then treated with actinomycin D. The RNA was harvested at the indicated time points (0, 4, 8, 12, 24 h), and the remaining levels of circZNF148 were quantified by qRT‑PCR and normalized to the 0 h time point. ns, no significance; *P < 0.05; **P < 0.01; ***P < 0.001.

**Figure S11. HK1 is identified as a key binding partner of circZNF148.** (A) Silver staining of proteins pulled down by circZNF148 or antisense oligonucleotide control. Mass spectrometry analysis identifies hexokinase 1 (HK1) as a top candidate circZNF148-interacting protein. (B) KEGG pathway enrichment of proteins identified in the HK1-associated complex. (C) Representative MS/MS spectra of two HK1-derived peptides. (D) RIP assay confirmed the endogenous circZNF148-HK1 interaction in BT-549 cells. (E) Mountain plot of circZNF148 RNA secondary structure and the schematic of the full-length and truncated mutants of circZNF148. (F) RNA pull-down assays confirmed the binding of HK1 to circZNF148 in BT-549 cells. ***P < 0.001.

**Figure S12. HK1 rescues the functional and metabolic defects caused by circZNF148 knockdown.** MDA-MB-231 and MDA-MB-468 cells were cotransfected with si-circZNF148 and HK1 overexpression vectors. (A) Transwell assays measuring cell migratory and invasive abilities. Scale bars, 200 μm. (B) Sphere formation assays measuring sphere formation capacity. Scale bars, 50 μm. (C) Flow cytometry analysis measuring ALDH⁺ proportions in MDA-MB-231 cells and CD44⁺CD24⁻ cancer stem-like subpopulations in MDA-MB-468 cells. (D-G) Intracellular glucose, G6P, ATP and lactic acid were measured. *P < 0.05; **P < 0.01; ***P < 0.001.

**Figure S13. circZNF148 stabilizes HK1 protein without affecting its transcription and reduces its ubiquitination.** (A) Effects of circZNF148 overexpression or knockdown on HK1 protein levels. (B) The effects of circZNF148 overexpression or knockdown on *HK1* mRNA levels. (C) Linear regression of normalized HK1 intensity showing the effects of circZNF148 overexpression or knockdown on HK1 half-life. (D) Following circZNF148 knockdown and treatment with 100 µg/mL CHX for indicated time, breast cancer cell lysates were subjected to western blot. Linear regression of normalized HK1 intensity showing the effects of circZNF148 knockdown on the half-life of HK1 protein. (E) Following circZNF148 knockdown, and treatment with MG132 (20 μM) for 5 h, breast cancer cell lysates were subjected to western blot. (F) Immunoprecipitation followed by immunoblotting shows the effects of circZNF148 overexpression on HK1 polyubiquitination in MDA-MB-231, MDA-MB-468, and BT-549 cells. ns, no significance.

**Figure S14. ADRM1 directly interacts with circZNF148 and HK1 to regulate HK1 stability.** (A) Representative MS/MS spectra of ADRM1-derived peptide and RNA pull-down assays confirming the direct binding between circZNF148 and ADRM1, but not UCH37. (B) RNA immunoprecipitation (RIP) confirming the direct binding between circZNF148 and ADRM1. (C) RNA pull-down assays confirming the direct binding between circZNF148 and ADRM1, but not UCH37. (D) Endogenous Co-IP confirming HK1-ADRM1 interaction. (E) Exogenous Co-IP confirming HK1-ADRM1 interaction. (F) IF showing HK1-ADRM1 colocalization. Scale bars, 20 μm. (G) Schematic of truncated ADRM1. (H) Truncation-based Co-IP mapping HK1-ADRM1 interaction to ADRM1 N-terminal domain. (I) RIP assay maps the middle domain of ADRM1 binding to circZNF148. ns, no significance; ***P < 0.001.

**Figure S15. circZNF148 facilitates HK1-ADRM1 interaction.** (A-B) Analysis of the HK1-ADRM1 interaction following circZNF148 overexpression by (A) endogenous co-immunoprecipitation (Co-IP) and (B) immunofluorescence (IF). (C-E) Analysis of the HK1-ADRM1 interaction following circZNF148 knockdown by (C) endogenous Co-IP, (D) IF, and (E) exogenous Co-IP. Scale bars, 20 μm. (F) Linear regression analysis of normalized HK1 intensity, assessing HK1 half-life upon ADRM1 overexpression or knockdown. *P < 0.05; **P < 0.01; ***P < 0.001.

**Figure S16. circZNF148 promotes the assembly of an ADRM1-UCH37-HK1 ternary complex.** (A) Endogenous Co-IP confirming ADRM1-UCH37 interaction. (B) Endogenous Co-IP confirming UCH37-HK1 interaction. (C) Endogenous immunoprecipitation followed by immunoblotting showing the effects of circZNF148 overexpression on UCH37-HK1 interaction. (D) Quantitative analysis of the co-localization between HK1 and UCH37 upon circZNF148 overexpression. (E) Truncation-based Co-IP mapping UCH37-ADRM1 interaction to ADRM1 C-terminal domain. (F) **3D molecular docking** **to visualize the spatial arrangement of the complex.** **P < 0.01; ***P < 0.001.

**Figure S17. circZNF148 shows no effects on the expression of ADRM1 or UCH37.** (A) The protein levels of ADRM1 and UCH37 are unaffected by circZNF148 overexpression or knockdown, as determined by western blot analysis. (B) The mRNA levels of *ADRM1 and UCH37* are unaffected by circZNF148 overexpression or knockdown, as determined by qRT-PCR analysis. ns, no significance.

**Figure S18. ADRM1 and UCH37 are functionally required for circZNF148-mediated oncogenic phenotypes.** (A-C) Ectopic expression of ADRM1 rescues the suppressed migration/invasion (Scale bars, 200 μm) (A), reduced stemness (Scale bars, 50 μm) (B), and impaired lactate production (C) caused by circZNF148 knockdown. (D-F) UCH37 overexpression reverses the deficits in migration/invasion (Scale bars, 200 μm) (D), stemness (Scale bars, 50 μm) (E), and lactate production (F) resulting from circZNF148 knockdown. *P < 0.05; **P < 0.01; ***P < 0.001.

**Figure S19. circZNF148 reprograms glycolysis to produce an immunosuppressive microenvironment.** (A) Flow cytometry analysis of isolated cells confirmed a high purity of CD8⁺ T cells following isolation from human PBMCs. (B) Glycolysis enhancement in metastatic cells and circZNF148 regulation. Glycolysis schematic (left). Lactate levels in parental vs. metastatic subline (middle). Effect of circZNF148 knockdown on lactate production (right). (C-D) Lactate production under circZNF148 overexpression with oxamate treatment (C) and circZNF148 knockdown with HK1 rescue (D). (E-F) Cytokine secretion (E) and gene expression (F) of CD8⁺ T cells exposed to conditioned media from circZNF148-overexpressing cells with or without oxamate treatment. (G) CFSE staining **showing the effect of 2-DG (20 mM) or oxamate (10 mM) on the proliferation of** CD8⁺ T cells. (H) **Annexin V/**7-AAD **assay detecting the effect of 2-DG or oxamate on the apoptosis of** CD8⁺ T cells. (I-J) Cytokine secretion (I) and gene expression (J) of CD8⁺ T cells treated with vehicle control, 2-DG, or oxamate. (K) Flow cytometry of PD‑1 and TIM‑3 expression of CD8⁺ T cells treated with vehicle control, 2-DG, or oxamate. ns, no significance; *P < 0.05; **P < 0.01; ***P < 0.001.

**Figure S20. circZNF148 regulates the function of CD8^+^ T cells.** (A-B) Cytokine secretion (A) and exhaustion marker expression (B) of CD8⁺ T cells exposed to conditioned media from control or circZNF148-knockdown cells. *P < 0.05; **P < 0.01; ***P < 0.001.

**Figure S21. Lactate anion suppresses CD8⁺ T cell function.** Previously activated and expanded human CD8⁺ T cells were cultured for 24 h under four conditions: standard medium (Ctrl), medium containing 20 mM sodium lactate (NaLa), an HCl-acidified control (HCl), and medium supplemented with 20 mM lactic acid (LA) (with the HCl condition pH-matched to the LA group). (A) CFSE staining analyzing the proliferation of CD8⁺ T cells. (B) Annexin V/7-AAD assays detecting the apoptosis of CD8⁺ T cells. (C) ELISA quantification of IFN‑γ and TNF‑α in culture supernatants of CD8⁺ T cells. (D) qRT-PCR analysis of gene expression of CD8⁺ T cells. (E) Flow cytometry analysis of PD‑1 and TIM‑3 expression on CD8⁺ T cells. ns, not significant; **P < 0.01; ***P < 0.001.

**Figure S22. circZNF148 regulates PD-L1 expression and membrane localization.** (A-C) The highly metastatic 231/Lung subline exhibits elevated PD-L1. (A) Immunoblot of PD-L1. (B) Flow cytometry analysis of surface PD-L1. (C) IF staining showing enhanced membrane localization of PD-L1 in 231/Lung cells. Scale bars, 20 μm. (D) Western blot analysis of total PD-L1 levels upon circZNF148 overexpression in TNBC cells. (E) Flow cytometry analysis of surface PD-L1 levels upon circZNF148 overexpression in TNBC cells. (F) Western blot analysis of total PD-L1 levels upon circZNF148 knockdown in TNBC cells. (G) Flow cytometry analysis of surface PD-L1 levels upon circZNF148 knockdown in TNBC cells. (H) IF staining showing reduced membrane localization of PD-L1 upon circZNF148 knockdown in TNBC cells. Scale bars, 20 μm. *P < 0.05; **P < 0.01; ***P < 0.001.

**Figure S23. circZNF148 modulates the global lactylated proteome via metabolic reprogramming.** (A) Global protein lactylation is elevated in 231/Lung cells and reduced upon circZNF148 knockdown (pan-lactyl-lysine immunoblot). (B) The pH of culture medium from MDA-MB-231 or MDA-MB-468 cells treated with vehicle control or sodium lactate (NaLa) for 24 h. (C) Western blot detection of global lactylation levels in control and circZNF148-knockdown TNBC cells cultured in the presence or absence of 20 mM NaLa for 24 h. (D) Immunoprecipitation of PD-L1 followed by immunoblotting with anti-lactyl-K antibody shows enhanced PD-L1 lactylation upon circZNF148 overexpression, which is attenuated by glycolytic inhibition. (E) Flow cytometry analysis shows the increased surface PD-L1 expression upon circZNF148 overexpression, which is attenuated by glycolytic inhibition. (F) Flow cytometry analysis shows the decreased surface PD-L1 expression upon circZNF148 knockdown, which is rescued by sodium lactate supplementation. (G) Immunoprecipitation assay evaluating PD-L1-specific lactylation in TNBC cells treated for 24 h with standard medium (Ctrl), medium supplemented with 20 mM NaLa, an HCl-acidified control (HCl), or medium containing 20 mM lactic acid (LA) (with the HCl condition pH-matched to the LA group). (H) Western blot analysis of global protein lactylation (Pan-Kla) in TNBC cells cultured under conditions described in (G). ns, not significant; **P < 0.01; ***P < 0.001.

**Figure S24. circZNF148 promotes metastatic potential in vivo.** (A) Quantification of TUNEL staining for apoptosis in tumor xenografts. (B) Quantification of IHC or ISH staining in tumor xenografts. (C) Representative images and quantification of lung metastatic nodules in mice following tail vein injection of control or circZNF148-overexpressing TNBC cells. Scale bars, 200 μm. ns, not significant; *P < 0.05; ***P < 0.001.
