# Supplementary material for: circZNF148 Drives Glucose Metabolism Reprogramming to Enhance Metastasis and Immune Evasion via HK1 Stabilization in Triple‐Negative Breast Cancer
Source: Adv Sci (Weinh). 2026 Aug 7;13(60):e77030. doi: 10.1002/advs.77030 (PMC13449536; doi:10.1002/advs.77030)
Supplement: Supplementary file 2 [file ADVS-13-e77030-s002.docx]

**Supplementary Tables**

**Table S1. siRNA used for transfection**

| Name | Sense (5’-3’) | Antisense (5’-3’) |
| --- | --- | --- |
| si-circZNF148 | GCAAAAGCAGUUAUACUUA | UAAGUAUAACUGCUUUUGC |
| si-ADRM1 | GUCUACGUGCUGAAGUUCA | UGAACUUCAGCACGUAGAC |
| si-UCH37 | CCACCAGGAUGUCCAUUUA | UAAAUGGACAUCCUGGUGG |
| si-IGF2BP2 | GGCAUCAGUUUGAGAACUA | UAGUUCUCAAACUGAUGCC |
| si-YBX1 | GGAGGCAGCAAAUGUUACA | UGUAACAUUUGCUGCCUCC |
| si-KHDRBS3 | CCUCAUCCCUGAUUAUAAU | AUUAUAAUCAGGGAUGAGG |
| si-NC | UUCUCCGAACGUGUCACGU | ACGUGACACGUUCGGAGAA |

**Table S2. Primer sequences**

| **Gene** | **Forward （5’-3’）** | **Reverse （5’-3’）** |
| --- | --- | --- |
| circZNF148 | GAAGAAGCGGAAACAACG | TCCACCCATTACAACCAT |
| HK1 | CTGCTGGTGAAAATCCGTAG | GTCCAAGAAGTCAGAGATG |
| ADRM1 | AGACGGACGACTCGCTTATT | ACTCACAGTCGTCAGGGAAG |
| UCH37 | TGTGGTTCAGGACTCCCGACTT | CGCCTAAATGGACATCCTGGTG |
| IGF2BP2 | GTTGGTGCCATCATCGGAAAGG | TGGATGGTGACAGGCTTCTCTG |
| YBX1 | GCAGGAGAACAAGGTAGACCAG | CTTCATTGCCGTCCTCTCTAGG |
| KHDRBS3 | GACATGGACTCAGTGAGGAGAC | TATGGCTGGTCTCTGTAGACGC |
| IFNG | GAGTGTGGAGACCATCAAGGAA | TGCTTTGCGTTGGACATTCAAG |
| PRF1 | ACTCACAGGCAGCCAACTTTG | CTCTTGAAGTCAGGGTGCAGC |
| GZMB | CGACAGTACCATTGAGTTGTGCG | TTCGTCCATAGGAGACAATGCCC |
| β-actin | CACTGTGCCCATCTACGAG | AATGTCACGCACGATTTCC |

**Table S3. Antibodies used in the experiments**

| **Antigen** | **Supplier** | **Catalog#** | **Application** |
| --- | --- | --- | --- |
| AKT | Beyotime | AF0045 | IB(1:1000) |
| ADRM1 | Proteintech | 11468-1-AP | IB(1:1000)IF(1:100) IHC(1:100) |
| ALDH1A1 | Invitrogen | MA5-29023 | IB(1:1000) |
| ALDOA | Proteintech | 11217-1-AP | IB(1:5000) |
| E-Cadherin | Selleck | F0005 | IB(1:1000) |
| Fibronectin | ABCAM | ab6328 | IB(1:1000) |
| Flag TAG | Beyotime | AF2852 | IB(1:1000) |
| Flag TAG | Invitrogen | PA1-984B | IB(1:1000) |
| GLUT1 | Selleck | F1192 | IB(1:1000) |
| HA TAG | ZSGB-BIO | TA-04 | IB(1:1000) |
| HA TAG | Thermo Fisher | PA1-985 | IB(1:1000) |
| HK1 | Proteintech | 15656-1-AP | IB(1:1000)IF(1:100) IHC(1:100) |
| HK2 | Proteintech | 22029-1-AP | IB(1:1000) |
| IGF2BP2 | Proteintech | 11601-1-AP | IB(1:1000) |
| Ikβ-a | Proteintech | 18220-1-AP | IB(1:1000) |
| Ki67 | Proteintech | 10197-1-AP | IHC(1:100) |
| LDHA | Selleck | F0233 | IB(1:1000) |
| L-Lactyl Lysine | PTM bio | PTM-1401 | IB(1:1000) |
| Myc TAG | ZSGB-BIO | TA-01 | IB(1:1000) |
| Myc TAG | Bethyl | A190-105A | IB(1:1000) |
| NANOG | Proteintech | 67255-1-Ig | IB(1:1000) |
| N-Cadherin | Affinity | af5239 | IB(1:1000) |
| NF-κB | Proteintech | 66535-1-Ig | IB(1:1000) |
| OCT4 | Proteintech | 11263-1-AP | IB(1:1000) |
| P-AKT | Proteintech | 66444-1-Ig | IB(1:1000) |
| PD-L1 | Proteintech | 66248-1-Ig | IB(1:1000)IF(1:100) IHC(1:100) |
| PFKP | Proteintech | 13389-1-AP | IB(1:1000) |
| p-Ikβ-a | CST | 2859T | IB(1:1000) |
| p-STAT3 | Affinity | AF3293 | IB(1:1000) |
| Slug | Selleck | F0362 | IB(1:1000) |
| Sodium Potassium  ATPase | Beyotime | AF1864 | IF(1:100) |
| SOX2 | Beyotime | AF8034 | IB(1:1000) |
| STAT3 | Affinity | AF6294 | IB(1:1000) |
| UCH37 | Proteintech | 68535-1-Ig | IF(1:100) |
| ZEB1 | Selleck | F0266 | IB(1:1000) |
| β-Actin | Proteintech | 66009-1-Ig | IB(1:1000) |
| β-CATENIN | Beyotime | AF1189 | IB(1:1000) |

**Table S4. Correlation between circZNF148 expression and clinicopathological characteristics of breast cancer patients.**

| **Characteristics** | **Total cases (n = 238)** | **circZNF148 expression level** | | |
| --- | --- | --- | --- | --- |
|  |  | **Low** | **High** | p value |
| **Age** |  |  |  |  |
| ≤ 50 | 123 | 69 | 54 |  |
| > 50 | 115 | 56 | 59 | 0.311136696 |
| **Menopausal state** |  |  |  |  |
| Pre | 115 | 66 | 49 |  |
| Post | 123 | 59 | 64 | 0.185175621 |
| **Pathological grade** |  |  |  |  |
| ≤ 2 | 163 | 84 | 79 |  |
| > 2 | 75 | 41 | 34 | 0.756608693 |
| **ER** |  |  |  |  |
| Positive | 153 | 86 | 67 |  |
| Negative | 85 | 39 | 46 | 0.163552508 |
| **PR** |  |  |  |  |
| Positive | 140 | 82 | 58 |  |
| Negative | 98 | 43 | 55 | 0.035531312* |
| **HER2** |  |  |  |  |
| Positive | 41 | 25 | 16 |  |
| Negative | 197 | 100 | 97 | 0.307869101 |
| **Ki67** |  |  |  |  |
| Low | 60 | 31 | 29 |  |
| High | 178 | 94 | 84 | 0.996993447 |
| **Tumor size** |  |  |  |  |
| ≤ 2 | 110 | 59 | 51 |  |
| > 2 | 128 | 66 | 62 | 0.84989581 |
| **Lymphatic metastasis** |  |  |  |  |
| Positive | 116 | 55 | 61 |  |
| Negative | 122 | 70 | 52 | 0.158930546 |
| **Metastasis status** |  |  |  |  |
| Positive | 34 | 12 | 22 |  |
| Negative | 204 | 113 | 91 | 0.046896024* |

**Table S5. Univariate and multivariate Cox regression analyses of overall survival.**

|  | **Univariate analysis** | | **Multivariate analysis** | |
| --- | --- | --- | --- | --- |
| **Index** | **HR (95% CI)** | ***p*** value | **HR (95% CI)** | ***p* value** |
| **Age** | 1.027(0.984-1.071) | 0.226 |  |  |
| **Menopausal status (pre vs. post)** | 0.751(0.297-1.904) | 0.547 |  |  |
| **Pathological Grade  (>G2 vs. G1&G2)** | 2.334(0.926-5.882) | 0.072 |  |  |
| **ER status  (ER+ vs. ER-)** | 0.641(0.253-1.626) | 0.349 |  |  |
| **PR status  (PR+ vs. PR-)** | 0.819(0.323-2.075) | 0.673 |  |  |
| **HER2 status  (HER2+ vs HER2-)** | 0.61(0.14-2.653) | 0.509 |  |  |
| **Ki67 positive rate  (High vs. low)** | 2.804(0.645-12.199) | 0.169 |  |  |
| **Tumor size** | 1.486(1.052-2.101) | 0.025* | 1.477(1.022-2.134) | 0.038* |
| **Lymph node status  (with metastasis vs.  without metastasis)** | 5.494(1.59-18.98) | 0.007* |  |  |
| **Metastasis status  (with metastasis vs.  without metastasis)** | 59.674(13.704-  259.856) | <0.001* | 51.022(10.950-237.340) | <0.001* |
| **circZNF148 expression  (High expression vs.  low expression)** | 1.206(1.047-1.388) | 0.009* | 1.228(1.031-1.462) | 0.021* |
